# The Neuropeptide PDF Is Crucial for Delaying the Phase of *Drosophila’s* Evening Neurons Under Long Zeitgeber Periods

**DOI:** 10.1177/07487304211032336

**Published:** 2021-08-24

**Authors:** Koustubh M. Vaze, Charlotte Helfrich-Förster

**Affiliations:** *Neurobiology and Genetics, Theodor-Boveri Institute, Biocenter, University of Würzburg, Würzburg, Germany; †Life Sciences Institute, University of Michigan, Ann Arbor, Michigan, USA

**Keywords:** *Drosophila*, circadian, PDF, entrainment, evening neurons, T-cycle, molecular clock

## Abstract

Circadian clocks schedule biological functions at a specific time of the day. Full comprehension of the clock function requires precise understanding of their entrainment to the environment. The phase of entrained clock is plastic, which depends on different factors such as the period of endogenous oscillator, the period of the zeitgeber cycle (T), and the proportion of light and darkness (day length). The circadian clock of fruit fly *Drosophila melanogaster* is able to entrain to a wide range of T-cycles and day lengths. Here, we investigated the importance of the neuropeptide Pigment-Dispersing Factor (PDF) for entrainment by systematically studying locomotor activity rhythms of *Pdf ^0^* mutants and wild-type flies under different T-cycles (T22 to T32) and different day lengths (8, 12, and 16 hour [h]). Furthermore, we analysed PERIOD protein oscillations in selected groups of clock neurons in both genotypes under T24 and T32 at a day length of 16 h. As expected, we found that the phase of *Drosophila’s* evening activity and evening neurons advanced with increasing T in all the day lengths. This advance was much larger in *Pdf ^0^* mutants (~7 h) than in wild-type flies causing (1) pronounced desynchrony between morning and evening neurons and (2) evening activity to move in the morning instead of the evening. Most interestingly, we found that the lights-off transition determines the phase of evening neurons in both genotypes and that PDF appears necessary to delay the evening neurons by ~3 h to their wild-type phase. Thus, in T32, PDF first delays the molecular cycling in the evening neurons, and then, as shown in previous studies, delays their neuronal firing rhythms to produce a total delay of ~7 h necessary for a wild-type evening activity phase. We conclude that PDF is crucial for appropriate phasing of *Drosophila* activity rhythm.

Circadian clocks allow animals to anticipate daily environmental changes by regulating various behaviours and physiological processes. Locomotor activity is one of them, and the circadian clock controlling activity rhythms in the fruit fly *Drosophila melanogaster* has been extensively characterized at the molecular and cellular level. The circadian activity rhythms in *Drosophila* are controlled by a network of about 150 neurons that are characterized by rhythmic expression of canonical clock genes (Helfrich-Förster, Shafer, et al., 2007; Hermann-Luibl and Helfrich-Förster, 2015; Top and Young, 2018). They are classified into 7 subgroups on the basis of their anatomical location and cell size as–small and large ventral lateral neurons (s-LN_v_s, l-LN_v_s), dorsal lateral neurons (LN_d_s), lateral posterior neurons (LPNs), and three groups of dorsal neurons (DN_1_s, DN_2_s and DN_3_s) (Helfrich-Förster, 2003; Helfrich-Förster, Yoshii, et al., 2007; Schubert et al., 2018). The daily activity of *Drosophila* under 12 h light/dark (LD 12:12) cycle exhibits bimodal patterns, comprising an activity peak in the morning (M), and one in the evening (E). The M and E activity peaks are regulated by distinct sets of lateral clock neurons, and genetic mosaic studies have shown that the s-LN_v_s are important for the M peak, whereas the 5^th^ LN (formerly known as 5^th^ s-LN_v_; Schubert et al., 2018) and the LN_d_ are important for the E peak (Grima et al., 2004; Stoleru et al., 2004, 2005, 2007; Rieger et al., 2006; Picot et al., 2007).

The 4 s-LN_v_s and 4 l-LN_v_s in each *Drosophila* brain hemisphere express the neuro-peptide Pigment Dispersing Factor (PDF) (Helfrich-Förster, 1995; Kaneko et al., 1997). PDF is necessary to maintain synchrony among clock neurons in DD (Peng et al., 2003; Lin et al., 2004); hence, PDF is regarded as a primary synchronizer of neuronal clocks that ensures the generation of coherent rhythmic output. Indeed, *Pdf* null mutants (*Pdf^0^*) usually lose circadian rhythmicity after a few days in DD and the few flies that remain rhythmic exhibit weak rhythms with moderate short period (~23 h) (Renn et al., 1999). Under LD 12:12, *Pdf^0^* mutants exhibit astonishingly stable activity-rest rhythms with reduced morning and slightly advanced E activity that may result from their short free-running period under DD (Renn et al., 1999). Furthermore, *Pdf^0^* mutants show robust molecular rhythms under LD 12:12 suggesting that loss of PDF does not cause severe behavioural or molecular rhythm defects under entrained conditions (Peng et al., 2003). However, Yoshii, Wülbeck, et al. (2009) demonstrated that *Pdf^0^* mutants fail to adjust their locomotor activity to changes in day length suggesting that PDF is crucial for seasonal adaptation. More recently, Liang et al. (2016) showed that PDF delays the Ca^2+^ rhythms in the E neurons and that it does so to a larger extent when the flies were kept under long days before recording. Again, this indicates that PDF is important for seasonal adaptation. As photoperiodic modulation of the daily activity profile depends on functional entrainment mechanisms, the failure of *Pdf^0^* mutants in adapting to changes in day length suggests that entrainment is defective in the absence of PDF.

We tested the importance of PDF for normal entrainment by systematically investigating locomotor activity rhythms of *Pdf^0^* mutants and wild-type *CantonS* flies under zeitgeber cycles with different periods and at three different day lengths. We increased zeitgeber periods (T) in 2 h steps from 22 h (T22) to 32 h (T32). This systematic experimental design is also known as ‘T-cycle approach’. Although, circadian clocks are adaptations to 24 h daily zeitgeber cycles, they are capable of entraining to T-cycles over a limited period range (Aschoff and Wever, 1962; Hoffmann, 1969). When circadian clock entrains to different T-cycles, the phase relationship of the overt rhythm with LD cycles (Ψ) systematically changes with T-cycle period (Roenneberg et al., 2003, 2005). Typically, Ψ of the rhythm delays in T < 24 h and advances in T > 24 h relative to Ψ in 24 h cycles (Aschoff and Wever, 1962; Hoffmann, 1969). Therefore, the T-cycle approach produces systematic relationships between zeitgeber period T and rhythm phase Ψ. The T-Ψ relationships are products of entrainment and depend on two key clock properties—(1) the endogenous period (τ), and (2) the phase dependent light responsiveness of the clock (characterized by the phase response curve (Johnson, 1992)). Consequently, differences in T-Ψ relationships indicate differences in the properties of entrainment.

*Drosophila’s* activity rhythms entrain to T-cycle periodicities ranging from 19 to 36 h, and, as explained above, M and E activity peaks occur later in T < 24 h and earlier in T > 24 h in comparison to T = 24 h (Helfrich and Engelmann, 1987; Helfrich-Förster, 2001). The goal of our experiments was to examine differences, if any, in the T-Ψ relationship between *Pdf^0^* mutants and wild-type flies, in case that PDF should be crucial for normal entrainment. To exclude that such putative differences are caused by the slightly shorter period of *Pdf^0^* mutants (τ~23 h), we also investigated the T-Ψ relationship of short-period mutants *period^short^* (*per^S^*) (τ~19 h) (Konopka and Benzer, 1971). Furthermore, to see whether the phase of the activity rhythm is reflected in the phase of the molecular clock, we analysed PERIOD protein (PER) oscillations in selected groups of clock neurons in *Pdf^0^* and wild-type flies under T24 and T32.

We found that *Pdf ^0^* mutants show drastically different T-Ψ relationships from wild-type flies and it cannot be solely explained by a shorter τ οf *Pdf^0^* flies. The phase advancing effect of entrainment to long T-cycle was so strong that under T32, E activity of *Pdf^0^* mutants occurred in the morning. The earlier phase of E activity was paralleled by an early phase of PER oscillations in the E neurons of *Pdf^0^* mutants under T32; their phase was advanced by about 3 h compared to the phase of the E neurons in wildtype. We conclude that PDF is needed for delaying the molecular clock (PER oscillations) in the E neurons to a wild-type phase, which in turn is essential for keeping E activity in second half of the day. Thus, under long days and especially under long zeitgeber periods, PDF delays the PER oscillations in the E neurons in addition to delaying their Ca^2+^ oscillations as previously shown by Liang et al. (2016, 2017). In addition, PDF is also necessary to delay the M neurons (s-LN_v_) under long zeitgeber periods. In summary, this confirms that PDF plays an important role in appropriate phasing of *Drosophila* activity rhythm and demonstrates that its role is more complex than thought before.

## Materials And Methods

### Fly Stocks

For recording locomotor activity rhythms, we used the wild-type strain *CantonS*, the PDF null mutant *Pdf* ^01^ (+; +; *Pdf ^0^*) (Renn et al., 1999; for simplicity just called *Pdf ^0^* mutants) and the short period mutant *per^S^* (Konopka and Benzer, 1971) that was backcrossed to wildtype for many generations (Horn et al., 2019). For immunostainings, we used a *Pdf ^0^* strain carrying a *Pdf*-RFP transgene (Ruben et al., 2012) (+; *Pdf-RFP; Pdf* ^o^), which has 0.6 kb of *Pdf* gene regulatory genomic DNA (0.5 kb upstream the start site of transcription and 0.1 kb downstream) fused to DNA encoding Red Fluorescence Protein mRFP1. For RNA interference (RNAi) knock-down of PDF expression, we used the strains–UAS-dicer2; *Pdf*-Gal4 and w;; UAS-*Pdf*-RNAi (BL 25802). The flies were reared under LD 12:12 cycles on standard cornmeal/agar medium at 25°C and 70% RH ± 5%. Only unmated, male flies of age 3 to 4 days were taken for the experiments.

### Recording the Locomotor Activity of Flies

Locomotor activity of individual male flies was recorded using the *Drosophila* activity monitoring system (Trikinetics Inc., Waltham, Massachusetts). Three- to four-day old unmated male flies were loaded into glass tubes (length 5 cm, diameter 5 mm) containing sugar-agar medium (4% sucrose, 2% agar in water). Flies were anesthetised with CO_2_ while collecting unmated males on the day of eclosion, and while loading the flies in activity recording tubes. Activity was recorded in 1 minute intervals under 15 different experimental LD cycle schedules which differed in two aspects–(a) period of LD cycle and (b) duration of light/ photoperiod per cycle. The LD cycles with periods ranging from 22 h to 32 h (T22, T24, T26, T28, T30, and T32) in combination with three different day lengths (8 h, 12 h, and 16 h) were used as described in Table 1. Light-dark schedule corresponding to each combination of T-cycle and day length is described in the respective box, e.g., LD 08:18 means LD cycle consisting of 8 h light and 18 h dark phase. The LD cycle schedules were implemented in light boxes fitted with white LED light sources; light intensity inside each box was set at 100 lux. Light boxes were housed in climate chamber with constant temperature, 20^o^C and 70% relative humidity ± 5%. Before starting the activity recording, all the flies were maintained on standard cornmeal/agar medium under rectangular LD 12:12 cycles at 20 °C and 70% RH ± 5%.

**Table 1.** LD schedules of T-cycles.

Photoperiod, h	T22	T24	T26	T28	T30	T32
8	LD 08:14	LD 08:16	LD 08:18	LD 08:20		
12	LD 12:10	LD 12:12	LD 12:14	LD 12:16	LD 12:18	
16	LD 16:06	LD 16:08	LD 16:10	LD 16:12	LD 16:14	LD 16:16

Abbreviation: LD = light/dark. The notation in each cell describes the duration of light and dark phases in hours for each T-cycle.

## Activity Data Analysis

### Average Activity Profile

Activity count data were binned into 15 min intervals. Actograms were plotted by specifying T-cycle periods using ActogramJ (Schmid et al., 2011). Flies were raised and maintained in LD12:12 before the beginning of activity recording, so both the genotypes showed several transient cycles before they stably entrained to the relevant T-cycle. Individual fly actograms were examined for stable entrainment under each T-cycle condition. The activity was recorded sufficiently long to obtain at least 6 cycles of stable entrainment at the end of activity recording in each T-cycle. In addition, the last six cycles of individual fly activity data from each T-cycle was analysed using Chi-square periodogram method (5% level of significance) in R package RhythmicAlly (Abhilash and Sheeba, 2019) to detect rhythmic flies and estimate period. Activity data from rhythmic flies were used for plotting activity profiles. For plotting activity profiles, data was first smoothened using 3-point moving average. The middle of the dark phase was chosen as the start-point of each cycle. Activity counts in each 15 min bin was normalized by dividing the count by total activity during the cycle and multiplying by 100. Each 15 min bin activity count was therefore expressed as percentage of total activity during the cycle. Average activity at each time-point (15 min interval) was calculated by taking average over 5 cycles within individual fly and over all the flies in the sample to plot an average activity profile of a genotype under each LD cycle.

### Phase of the Activity Rhythm

The phase relationship of activity rhythm with light-dark cycle—Ψ, was estimated using E peak as a phase marker. Timing of E activity peak for each fly was estimated by manually identifying the time-point showing maximum normalized activity level in the average activity profile. Average timing of E peak for a genotype under particular LD cycle was estimated by taking the average over estimates from all the flies in a sample. Phase of the E activity peak was either expressed in hours (h) after lights-on (corresponding to zeitgeber Time [ZT]); ZT00 is the time of lights-on) or in hours after lights-off. The effect of T-cycle period on Ψ was tested by performing a Kruskal-Wallis test on each genotype separately. The statistical significance of difference in Ψ between genotypes under any given T-cycle was tested using Mann-Whitney U test.

### Immunocytochemistry

Flies were maintained in T24 (LD 16:8) and T32 (LD 16:16) for 15 days to ensure stable entrainment to T32 and on 16th day whole flies were quickly fixed in 4% paraformaldehyde in phosphate buffer (PB) with 0.1% Triton X-100 for a subsequent 2.5 h at room temperature. For immunohistochemistry on whole-mount brains, the fixed flies were rinsed 3 times in PB and the brains dissected in PB. The brains were blocked overnight in 5% normal goat serum (NGS) at 4 °C and subsequently incubated at 4 °C for 48 h in primary antibodies diluted in PB containing 5% NGS and 0.5% Triton X-100. Wildtype brains were stained with rabbit anti-PER (1:2000) and mouse anti-PDFc7 (1:2000), and *pdf-RFP; Pdf* ^0^ brains were stained with rabbit anti-PER (1:2000) and rat anti-mCherry (1:2000). Secondary fluorescence-conjugated antibodies diluted 1:200 were applied for 3 h at room temperature following washing of 6 times in PB with 0.5% Triton X-100. Wildtype brains were incubated with Alexa Fluor 635 (goat anti-rabbit) and Alexa Fluor 488 (goat anti-mouse) (Molecular Probes, Carlsbad, CA); and *Pdf-RFP; Pdf* ^0^ brains were incubated with Alexa Fluor 635 (goat anti-rabbit) and Alexa Fluor 555 (goat anti-rat) secondary antibodies. After the incubation in the secondary antibodies, the brains were washed 6 times in PB with 0.5% Triton X-100 and mounted in Vectashield mounting medium (Vector Laboratories, Burlingame, CA).

### Image Acquisition and Analysis

For quantification of fluorescence signal from PER staining, whole mount brains were imaged by using a Leica TCS SP8 laser confocal microscope (Leica Microsystems, Wetzlar, Germany). Confocal stacks consisting of 2 μm thick optical sections were taken at 400 Hz using the 635 nm laser to visualize PER (Alexa Fluor 635) and 488 nm laser to visualize PDF (Alexa Fluor 488) or 555 nm laser to visualize RFP (Alexa Fluor 555). All brains were processed similarly, and the same settings were kept for all of the scans, time points, and genotypes. Images were analysed to quantify the intensity of PER protein levels using tools in ImageJ in 8 to 10 hemispheres from separate brain samples per genotype and time point. Mean nuclear PER staining intensity of different cell groups in each brain sample was estimated by measuring mean intensity in an arbitrarily chosen square-shaped area of 3 × 3 pixels from the brightest focal plane in individual cells. The large number of very small, tightly packed cells in the DN_3_ group of neurons makes it difficult to quantify staining intensity in individual neurons. Therefore, we obtained maximum intensity projection over 10 consecutive slices from the brain region covering DN_3_ neurons. The mean pixel intensity of DN_3_ neurons was estimated as average of mean pixel intensities from three square shaped areas (10 × 10-pixel) spanning the DN_3_ cluster. Intensity was measured in grayscale units, ranging between 0 (black; no staining) and 255 (white; saturated staining). The background staining was measured similarly. After correcting for background, replicate PER intensity values were plotted against the sample collection time-point (time since lights-off, h) for each neuronal subgroup and genotype. Rhythmicity in nuclear PER abundance time series was analysed using single component COSINOR based method by fitting cosine wave function of a specified period (24 or 32 h) to PER intensity data (Cornelissen, 2014). COSINOR analysis was implemented using CATcosinor function from the CATkit package written in R (Lee Gierke and Cornelissen, 2016).

## Results

### Entrainment of *Pdf^0^* Mutants Is Different From That of Wildtype Flies

We hypothesized that if the mechanism of light entrainment is defective in *Pdf ^0^* mutants, the T-Ψ relationship will differ between *Pdf ^0^* and wild-type flies. We tested this hypothesis by studying the activity-rest rhythms of the two strains under T-cycles with periods between 22 and 32 h. T-cycle entrainment studies typically set day length as a fixed percentage of T-cycle period. As a result, the absolute duration of the light phase changes with T-cycle period, causing the zeitgeber strength to differ between T-cycles. To avoid such a variation in zeitgeber strength, we kept the day length constant throughout the T-cycles (Table 1), but we performed the entire T-cycle experiments under three different day lengths (8 h, 12 h, and 16 h). This allowed testing for effects of day length separately from the effects of zeitgeber period on the T-Ψ relationship.

Wild-type and *Pdf ^0^* flies were raised and maintained in LD12:12 before the beginning of activity recording in T-cycles, so both the genotypes showed several transient cycles before they stably entrained to the T-cycle regimes. Visual inspection of individual fly actograms revealed clear evidence for stable entrainment, at least during the last 6-8 days of recording in each T-cycle regime in both the genotypes. Moreover, Chi-square periodogram analysis of the last 6 cycles of activity data under T-cycles showed a good match between rhythm period and the period of the entraining T-cycles in wild-type and *Pdf ^0^* flies (Table 2).

**Table 2.** Percent rhythmicity, period and power of activity rhythms in wildtype, *Pdf^0^* and *per^S^* flies under T-cycle entrainment.

	T-Cycle	*n*	*n* Rhythmic (%)	Period (SEM), h	Power (SEM)	n	Phase of Evening Peak (SEM) ZT, h
wildtype 8 h	T22	31	31 (100)	22.00 (0.00)	101.01 (9.34)	31	8.83 (0.21)
	T24	31	31 (100)	24.04 (0.02)	117.05 (9.15)	31	8.30 (0.04)
	T26	30	30 (100)	26.00 (0.00)	165.67 (13.05)	30	7.24 (0.11)
	T28	30	20 (93.33)	28.02 (0.02)	136.13 (12.75)	27	6.66 (0.13)
*Pdf^0^* 8 h	T22	31	31 (100)	21.96 (0.02)	116.42 (9.43)	31	7.24 (0.12)
	T24	29	29 (100)	23.98 (0.02)	169.25 (10.71)	29	5.71 (0.18)
	T26	31	31 (100)	25.95 (0.02)	139.01 (10.00)	28	4.44 (0.20)
	T28	31	31 (100)	28.02 (0.03)	163.36 (14.63)	31	2.56 (0.16)
wildtype 12 h	T22	31	29 (93.55)	22.00 (0.00)	129.55 (12.07)	29	12.02 (0.12)
	T24	28	25 (89.29)	23.99 (0.01)	157.32 (17.34)	24	11.78 (0.12)
	T26	29	29 (100)	26.00 (0.00)	164.11 (13.06)	28	11.29 (0.08)
	T28	31	29 (93.55)	28.01 (0.01)	147.64 (16.55)	28	10.61 (0.11)
	T30	29	29 (100)	30.10 (0.05)	132.06 (14.39)	27	9.04 (0.15)
*Pdf^0^* 12 h	T22	32	30 (93.75)	21.99 (0.01)	128.28 (12.32)	29	10.49 (0.15)
	T24	30	29 (96.67)	23.93 (0.04)	146.51 (11.5)	28	8.35 (0.20)
	T26	32	27 (84.38)	25.94 (0.04)	150.14 (14.15)	27	6.71 (0.25)
	T28	26	24 (92.31)	27.91 (0.05)	115.11 (12.47)	23	5.18 (0.24)
	T30	23	23 (100)	29.93 (0.05)	186.09 (17.37)	23	2.38 (0.25)
*per^S^* 12 h	T22	31	28 (90.32)	21.99 (0.01)	160.04 (14.47)	28	9.93 (0.12)
	T24	28	27 (96.43)	24.00 (0.01)	174.72 (13.95)	28	8.48 (0.11)
	T26	30	28 (93.33)	26.00 (0.03)	122.61 (14.31)	25	7.60 (0.12)
	T28	30	26 (86.67)	28.00 (0.02)	121.04 (14.93)	26	5.71 (0.11)
	T30	30	27 (90.00)	30.03 (0.03)	114.59 (15.76)	27	3.43 (0.14)
wildtype 16 h	T22	31	31 (100)	22.00 (0.00)	254.39 (15.38)	31	15.31 (0.07)
	T24	31	31 (100)	24.00 (0.00)	209.15 (13.68)	31	14.81 (0.12)
	T26	30	30 (100)	26.00 (0.00)	225.76 (15.9)	30	14.35 (0.13)
	T28	27	27 (100)	28.00 (0.00)	224.56 (18.13)	27	13.10 (0.20)
	T30	30	29 (96.67)	30.01 (0.02)	136.52 (14.72)	26	12.69 (0.21)
	T32	30	30 (100)	32.00 (0.02)	259.54 (20.98)	28	10.58 (0.16)
*Pdf^0^* 16 h	T22	31	28 (90.32)	21.94 (0.03)	107.94 (13.84)	28	13.06 (0.20)
	T24	29	27 (93.10)	23.94 (0.03)	188.49 (15.08)	27	11.48 (0.23)
	T26	31	30 (96.77)	25.98 (0.03)	119.65 (12.27)	30	9.98 (0.14)
	T28	29	29 (100)	27.95 (0.04)	121.93 (9.19)	29	8.02 (0.23)
	T30	31	31 (100)	29.97 (0.05)	168.79 (13.74)	31	5.81 (0.21)
	T32	29	29 (100)	31.95 (0.05)	162.58 (13.5)	27	3.25 (0.18)

Abbreviations: SEM = Standard Error of Mean; ZT = zeitgeber Time. Six cycles of activity data from individual fly under T-cycle entrainment was analysed using Chi-square periodogram method (5% level of significance) to test rhythmicity and estimate period. Data from rhythmic flies was further analysed to estimate phase of E activity peak.

**Figure 1.**
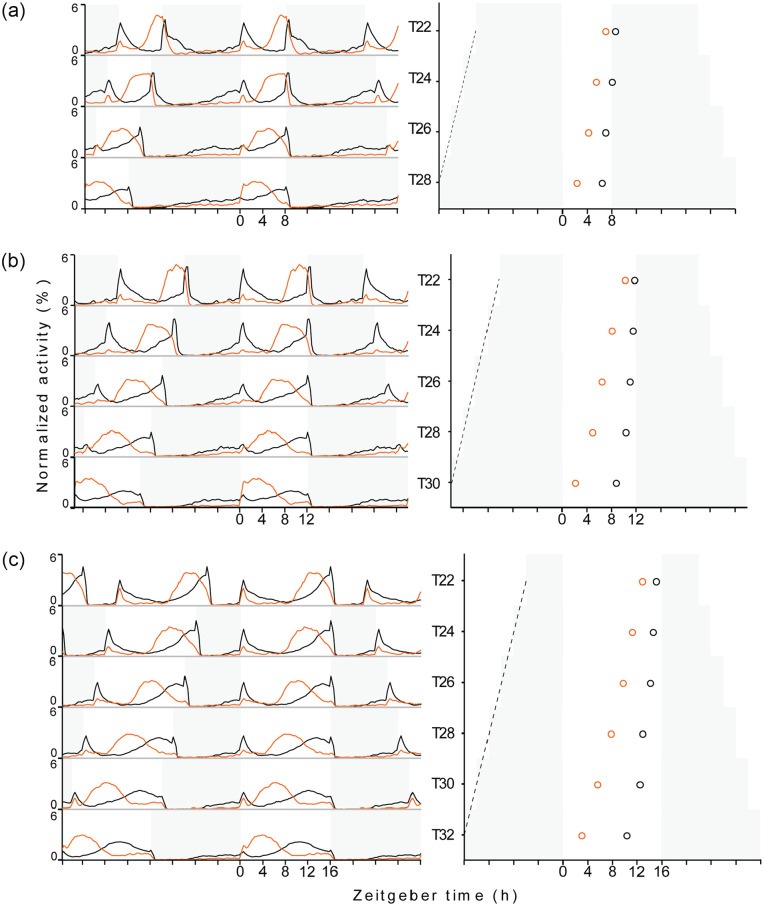
Activity-rest profiles and the phases of E activity peak in wildtype and *Pdf ^0^* mutants entrained to T-cycles. (a-c) show average activity-rest profiles of flies entrained to T-cycles in the left panel and phases of E activity peak in the right panel. a, b and c represent T-cycle sets with 8, 12 and 16 h photoperiod respectively, with T-cycle period increasing from top to bottom. White and grey regions depict light and dark phases respectively. Left panels: The black and orange line plot under each T-cycle depicts average normalized activity profile generated from activity recordings of 23-32 wildtype and *Pdf ^0^* mutant flies respectively. Activity level at each time point is an activity over 15 minute interval expressed as a percentage of total activity during one complete LD cycle (midnight to midnight), averaged over 5 days within each fly and over total number of flies in a sample. Right panels: Black and orange open circles depict the average phase of E activity peak in wildtype and *Pdf^0^* mutant flies respectively, estimated from E activity peak phases of 23-32 flies. The difference between E activity peak phases of wildtype and *Pdf^0^* mutant flies were tested for significance by pairwise Mann Whitney U test; differences are significant under all the LD cycles (Table 5). The dashed black line depicts line joining lights-off transitions of the preceding LD cycles over T-cycle period range. Abbreviation: LD = light/dark.

In all the light regimes, wild-type flies exhibited bimodal activity patterns with M and E activity peaks; whereas *Pdf ^0^* mutants showed prominent E activity peaks and highly reduced M activity, which is typical for these mutants. We therefore focused our analysis on E activity and used the timing of the E peak to assess the phase of the rhythm. As expected, both strains showed systematic changes in rhythm phase with the increasing period of the entraining T-cycles under all the three day-lengths. Relative to the timing under 24 h cycles, E peaks delayed in T < 24 h and gradually advanced in T > 24 h. In short, over the range of T-cycles tested, E peak gradually advanced with the increasing zeitgeber period of the entraining LD-cycles in both the genotypes (Figure 1; Table 2; Suppl. Figs. S1-S3; Suppl. Figs. S4-A-S4-C). This is exactly what we expected and it indicates that the activity rhythms of the two genotypes stably entrained to the T-cycles tested in our study.

Most interestingly, the phase of E activity depended additionally on day length (Figure 1). It got delayed with increasing day length and this seemed again true for both genotypes. A three factor ANOVA testing for the effects of zeitgeber period, day length and genotype on the phase of E activity (done for T22 to T28) revealed highly significant effects of all factors on the timing of E activity, zeitgeber period: F_(3,663)_ = 496.96; *p* < .001; day length: F_(2,663)_ = 2943.38; *p* < .001; genotype: F_(1,663)_ = 2716.92; *p* < .001. Furthermore, this ANOVA revealed significant interactions between genotype and zeitgeber period, F_(3,663)_ = 96.67; *p* < .001, and between genotype and day length, F_(2,663)_ = 24.70; *p* < .001, meaning that the effect of zeitgeber period and day length on the rhythm phase was genotype dependent.

**Figure 2.**
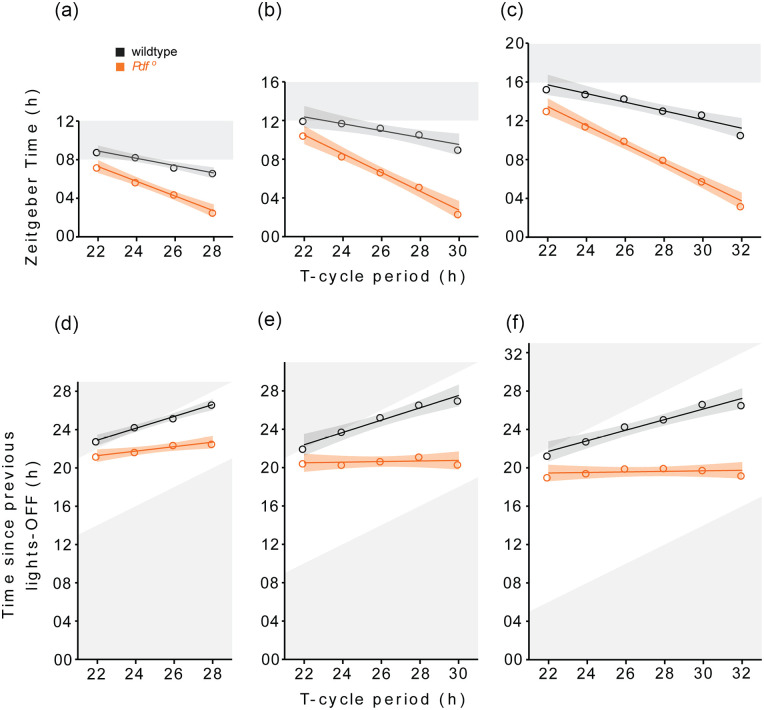
T-Ψ relationships in wildtype and *Pdf ^0^* flies. T-Ψ relationships of wildtype and *Pdf ^0^* flies under 8 h (a, d), 12 h (b, e) and 16 h (c, f) photoperiod conditions. Black and red open circles represent the average phases of E activity peak in wildtype and *Pdf* ^0^mutant flies plotted as a function of T-cycle period. Solid lines represent best fitting linear relationships obtained by linear regression and shaded ribbons depict standard errors. The phases of E activity peak are expressed as time since lights-on (h) in a, b and c; and time elapsed since previous lights-off (h) in d, e and f.

Significant effects of the zeitgeber period on the E peak phases of wild-type and *Pdf ^0^* flies were also revealed by the Kruskal-Wallis tests (this time we included all zeitgeber periods from T22 to T32 in the test, Table 3). Between the shortest and longest T-cycle periods, the E peaks of wild-type flies advanced by ~ 2 h, 3 h, and 5 h under the 8 h, 12 h, and 16 h day lengths, respectively. In *Pdf ^0^* mutants, the advance was almost twice as large as in wild-type flies and amounted ~ 4.5 h, 8 h, and 10 h, respectively (Figure 1; Table 2). Consistently, the linear regression showed that slopes of the T-Ψ relationship were steeper for *Pdf ^0^* mutants than for wild-type flies (Figure 2a-2c; Table 4[A]) showing that E peak in *Pdf ^0^* is advanced by much larger magnitude than in the wild-type for every hour rise in T-cycle period.

**Table 3.** Summary of Kruskal-Wallis tests on the E activity peak phases across T-cycles in wildtype, *Pdf^0^* and *per^S^* mutant flies.

Photoperiod	Genotype	Groups	H	*p*-value
8 h	wildtype	4	93.66	**<**.0001
	*Pdf^0^*	4	95.35	<.0001
12 h	wildtype	5	100.40	<.0001
	*Pdf^0^*	5	111.10	<.0001
	*per^S^*	5	122.30	<.0001
16 h	wildtype	6	135.30	<.0001
	*Pdf^0^*	6	155.80	<.0001

**Table 4.** Summary of linear regression analysis.

Photoperiod	Genotype	Slope	*R* ^2^	*p*-Value
A				
8 h	Wildtype	−0.37	0.98	<.01
	*Pdf^0^*	−0.76	0.99	<.01
12 h	Wildtype	−0.35	0.88	<.05
	*Pdf^0^*	−0.96	0.98	<.01
	*per^S^*	−0.78	0.97	<.01
16 h	Wildtype	−0.44	0.92	<.01
	*Pdf^0^*	−0.97	0.98	<.001
B				
8 h	Wildtype	0.62	0.99	<.01
	*Pdf^0^*	0.23	0.94	<.05
12 h	Wildtype	0.64	0.96	<.01
	*Pdf^0^*	0.03	0.07	Ns
	*per^S^*	0.21	0.72	.06
16 h	Wildtype	0.55	0.95	<.01
	*Pdf^0^*	0.03	0.07	Ns

The slope, R^2^ value and *p*-value of the linear relationship between T-cycle period and phase of E activity peak in wildtype, *Pdf ^0^* and *per^S^* flies under different photoperiod conditions. Summaries of regression analysis performed (A) using E activity peak phases expressed as time since lights-on (h), and (B) using E activity peak phases expressed as time elapsed since previous lights-off (h).

We also studied the activity-rest rhythms of flies where PDF expression was knocked down in s-LN_v_s and l-LN_v_s using RNA interference (RNAi) strategy, under T24 and T30 (16 h daylength). The PDF RNAi knock-down also advanced the E peak phase under T24 and T30, but not as much as in *Pdf ^0^* mutants (Suppl. Figs. S5A and S5B; Suppl. Table S6). The PDF RNAi knock-down flies lacked PDF staining in the arborizations of the s-LN_v_s and showed strongly reduced staining in that of the l-LN_v_s, but staining was still visible in the cell bodies suggesting an incomplete knock-down of PDF expression (Suppl. Fig. S5 C). The incomplete knock-down of PDF explains the smaller phase advance of the E peak in PDF RNAi knock-down flies. These results support our observations in *Pdf ^0^* mutants, and suggests that the large phase advance of the E peak in *Pdf ^0^* mutants under long T-cycles stems from the loss of PDF, and is not caused by different genetic backgrounds. All these observations clearly indicate that the entrainment properties of *Pdf ^0^* mutants are different from that of wild-type flies.

### Evening Activity Peak in *Pdf^0^* Appears to Follow the Previous Lights-Off

A closer examination of the data suggests that T-Ψ relationship in *Pdf ^0^* mutants runs almost parallel to a straight line drawn through the lights-off transitions of the preceding LD cycles (dashed black lines in right panels of Figure 1a-1c). Therefore, we plotted T-Ψ relationship by expressing the phases of E activity peak as time since previous lights-off instead of the time that passed since lights-on (Figure 2d-2f); and tested if the T-Ψ relationships ran parallel to the X-axis and if the slopes were equal to zero. The linear regression showed that the phase of E activity peak gradually delayed relative to the previous lights-off transition in wild-type flies as period of the T-cycle lengthened, producing positive slopes which were significantly different from zero (Figure 2d-2f; Table 4[B]). In *Pdf ^0^* mutants, on the other hand, the slope of the T-Ψ relationship was only significantly different from 0 under the 8 h photoperiod (here it amounted to 0.23). Under the 12 h and 16 h photoperiod, slopes were 0.03 and 0.02, respectively, and not significantly different from 0 (Figure 2d-2f; Table 4[B]). This analysis confirms that the activity peak in *Pdf ^0^* mutants tend to lag the lights-off transition by a constant time interval, suggesting that the clock controlling activity rhythm in *Pdf ^0^* mutants is driven by the light-dark cycle. Nevertheless, the constant time interval between lights-off and the peak of E activity was different for the three different day lengths.

### T-Ψ Relationships of *per^S^* and *Pdf^0^* Mutant Flies

**Figure 3.**
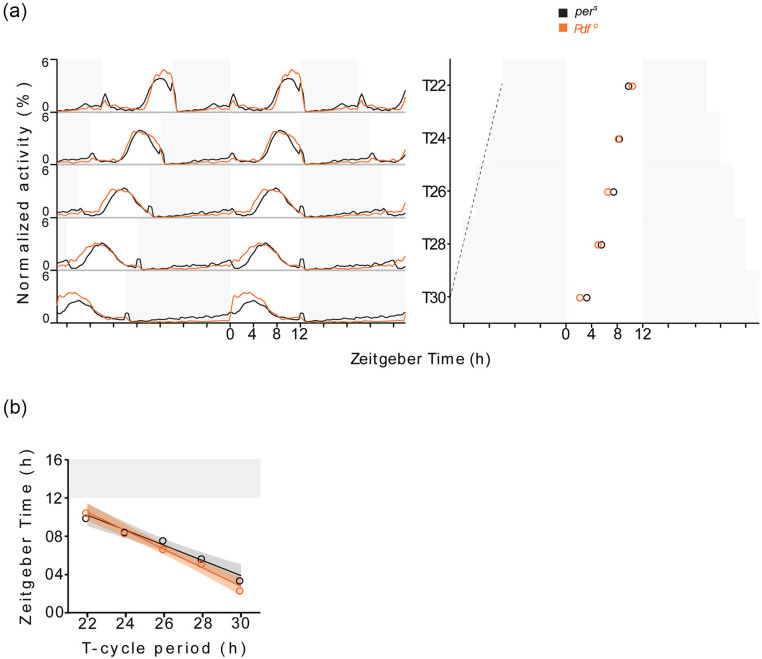
Activity rhythms of *per^S^* and *Pdf ^0^* mutants under T-cycle entrainment. (a) Activity profiles (left) and phases of E activity peak (right) of *per^S^* and *Pdf*^0^ mutants under T-cycles with 12 h photoperiod. Black and red line plot under each T-cycle in left panel depicts average normalized activity profile estimated from activity recordings of 23-29 *per^S^* and *Pdf ^0^* flies respectively. Activity level at each time point is an activity over 15 minute interval expressed as a percentage of total activity during one complete LD cycle (midnight to midnight), averaged over 5 days within each fly and over total number of flies in a sample. Black and red open circles in right panel depict the average phases of E activity peak in *per^S^* and *Pdf^0^* flies respectively, estimated from E activity peak phases of 23-29 flies. The dashed black line depicts line joining lights-off transitions of the preceding LD cycles over T-cycle period range. (b) T-Ψ relationships of *per^S^* and *Pdf*^0^ mutants. Black and red open circles represent the average phases of E activity peak in *per^S^* and *Pdf*^0^ mutant flies plotted as a function of T-cycle period. Solid lines represent best fitting linear relationships obtained by linear regression and shaded ribbons depict standard errors. Abbreviation: LD = light/dark.

We studied activity rhythm of short period mutant *per^S^* (τ = 19 h; Konopka and Benzer, 1971) under T-cycles with 12 h photoperiod to analyze the contribution of a short endogenous period to the T-Ψ relationship. As mentioned above, *Pdf ^0^* mutants also have a shorter τ (23 h) than wild-type flies and though it is only 1 h shorter than that of wild-type flies, this can affect the T-Ψ relationship. Like wild-type flies and *Pdf ^0^* mutants, *per^S^* mutants significantly advanced the E activity peak with increasing zeitgeber period (Figure 3a; Table 3). Between T22 and T30, the phase of E activity peak advanced by ~ 8 h *Pdf ^0^* mutant, whereas it advanced by ~ 6.5 h in *per^S^* (Figure 3a; Table 2). The linear regression analysis showed a T-Ψ relationship with negative slope that is similar to but less steep than in *Pdf ^0^* mutants (Figure 3b; Table 4[A]). Moreover, the phases of E activity peak were significantly different between *per^S^* and *Pdf ^0^* mutants under T22 and T30 (Table 5). Together these observations indicate that the phase advance under T-cycle entrainment in *Pdf ^0^* mutants is larger than in *per^S^* mutants, although *per^S^* mutants have a shorter period than *Pdf ^0^* mutants. Thus, the short τ alone cannot explain the differences in T-Ψ relationship between wild-type flies and *Pdf ^0^* mutants.

**Table 5.** Summary of Mann–Whitney U tests to compare the E activity peak phases between genotypes under T-cycle entrainment.

Genotype/Photoperiod	T-Cycle	U	*p*-Value
wildtype–*Pdf^0^* 8 h	T22	74	<.0001
	T24	0.5	<.0001
	T26	6.5	<.0001
	T28	1	<.0001
wildtype–*Pdf^0^* 12 h	T22	62	<.0001
	T24	4	<.0001
	T26	0	<.0001
	T28	0	<.0001
	T30	0	<.0001
wildtype–*Pdf^0^* 16 h	T22	12	<.0001
	T24	6.5	<.0001
	T26	0	<.0001
	T28	1.5	<.0001
	T30	0	<.0001
	T32	0	<.0001
*per^S^- Pdf*^0^ 12 h	T22	226	.0034
	T24	335.5	.3536
	T26	193.5	.0075
	T28	206	.0611
	T30	125.5	.0002

### Molecular Clocks of Wild-Type and *Pdf^0^* Mutant Flies Entrained to T24 and T32

**Figure 4.**
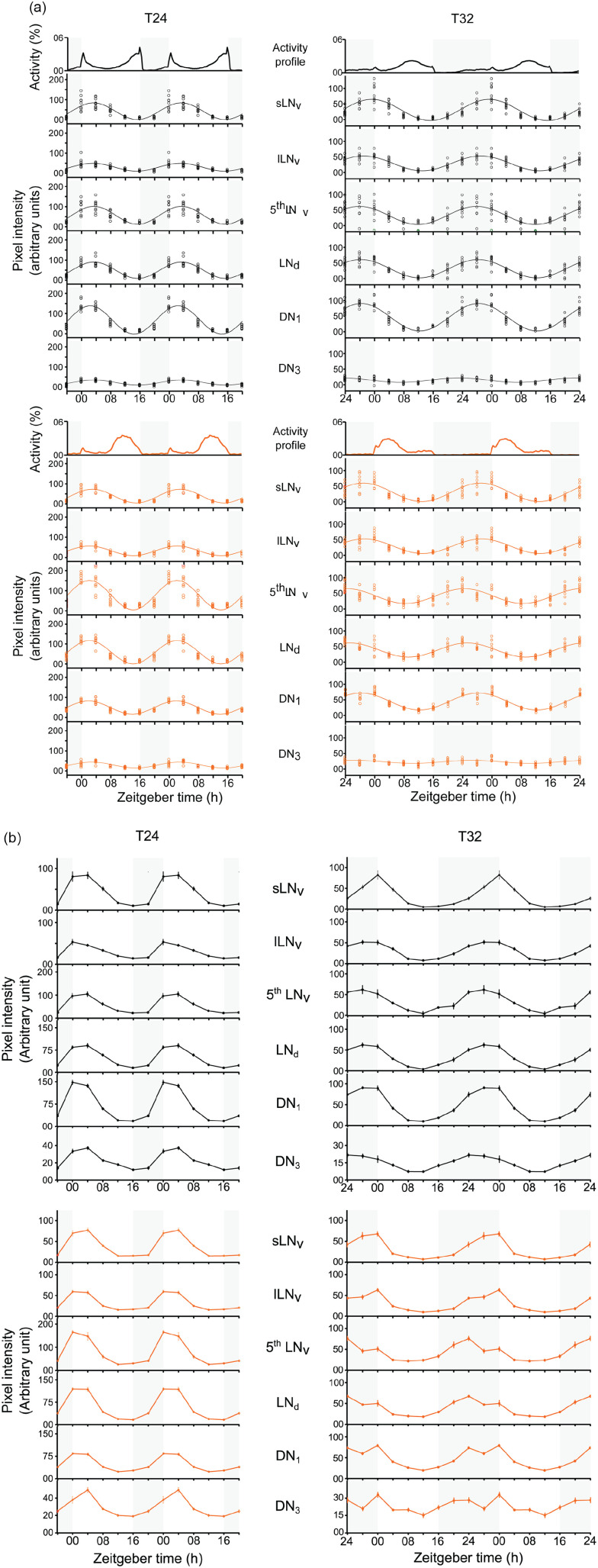
PER rhythms in clock neurons of wildtype and *Pdf ^0^* flies. (a) Scatter plots of background corrected nuclear PER intensities in clock neurons of wildtype (upper panels) and *Pdf ^0^* mutant (lower panels) flies entrained to T24 and T36. Each open circle represents the mean PER staining intensity in a clock neuron group estimated from replicate brains in a sample. The solid line depicts the best-fitting COSINE curve obtained by single-component COSINOR analysis. (b) Line plots of mean nuclear PER intensity plotted as a function of time after lights-on (h) in clock neurons of wildtype (upper panels) and *Pdf ^0^* (lower panels) flies entrained to T24 and T36. The staining intensity represents an average over intensity estimates from replicate brain tissues in a sample. Error bars are Standard Error of Means. Abbreviations: PER = PERIOD protein; s-LN_v_s = small lateral ventral neurons; l-LN_v_s = large lateral ventral neurons; LN_d_s = lateral dorsal neurons; DN_1_ = dorsal neurons 1; DN_3_ = dorsal neurons 3.

**Figure 5.**
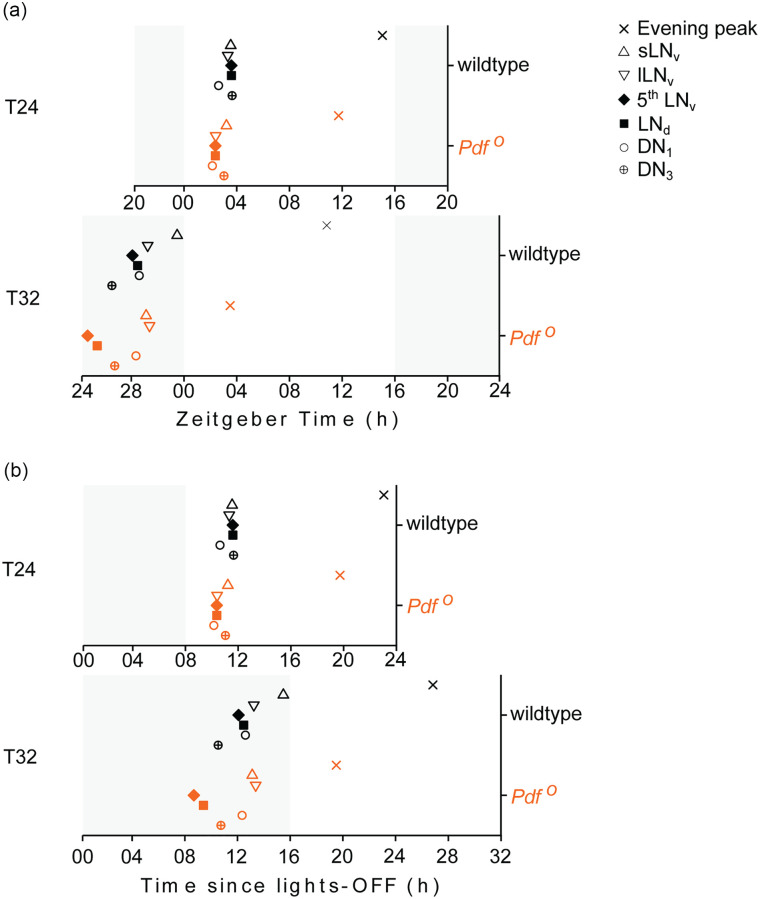
Phases of PER abundance peaks in clock neurons. Phases of nuclear PER abundance peaks in clock neurons relative to the phase of E activity peak in wildtype and *Pdf^0^* flies entrained to T24 and T32. The phases of PER peaks in individual clock neuron groups were estimated using single-component COSINOR based method. The phases are expressed in Zeitgeber time (h) units in a, and as time elapsed since lights-off transition of the preceding LD cycle (h) in b. Abbreviations: PER = PERIOD protein; s-LN_v_s = small lateral ventral neurons; l-LN_v_s = large lateral ventral neurons; LN_d_s = lateral dorsal neurons; DN_1_ = dorsal neurons 1; DN_3_ = dorsal neurons 3.

To test whether the large phase difference in E activity between *Pdf ^0^* mutants and wild-type flies under T32 is reflected in the phase of the molecular clock, we performed immunostainings against the clock protein PER. Wild-type flies and *Pdf ^0^* mutants were entrained to 24 h (LD 16:8) and 32 h (LD 16:16) LD cycles and their brains stained every 4 hours. We focused our analysis on nuclear PER staining intensities in six groups of clock neurons (s-LN_v_s, l-LN_v_s, 5^th^ LN, LN_d_s, DN_1_s and DN_3_s). Under both cycles, all clock neuron groups in wild-type flies and *Pdf ^0^* mutants showed visible PER rhythms, mostly with one peak (Figure 4a). Single-component COSINOR analysis revealed that PER-cycling was statistically significant in all the neuronal groups (Table 6). The COSINOR analysis also estimated the phase of PER peak for each clock neuron group (Figure 5a; Table 6), however the statistical significance of difference between the phases of neurons could not be ascertained as the data come from a single immunostaining experiment.

**Table 6.** Contribution of the 24 h or 32 h periodicity, their statistical significance and timings of peak phases of nuclear PER in clock neurons.

	PR	*p*-Value	Peak (*SE*)	PR	*p*-Value	Peak (*SE*)
			ZT, h			ZT, h
	T24			T32		
Wildtype						
sLN_v_	68.81	<.001	3.29 (0.34)	67.67	<.001	31.23 (0.41)
lLN_v_	60.18	<.001	3.07 (0.41)	74.00	<.001	28.99 (0.35)
5th LN_v_	67.77	<.001	3.34 (0.35)	50.66	<.001	27.83 (0.58)
LN_d_	80.71	<.001	3.35 (0.25)	77.94	<.001	28.22 (0.31)
DN_1_	84.70	<.001	2.37 (0.21)	85.68	<.001	28.35 (0.23)
DN_3_	70.91	<.001	3.40 (0.33)	52.84	<.001	26.27 (0.56)
*Pdf^0^*						
sLN_v_	78.06	<.001	2.97 (0.27)	66.61	<.001	28.87 (0.42)
lLN_v_	73.79	<.001	2.15 (0.30)	70.37	<.001	29.13 (0.38)
5th LN_v_	72.08	<.001	2.13 (0.31)	56.26	<.001	24.44 (0.52)
LN_d_	84.28	<.001	2.13 (0.22)	67.03	<.001	25.16 (0.41)
DN_1_	82.34	<.001	1.90 (0.23)	79.73	<.001	28.10 (0.29)
DN_3_	64.53	<.001	2.78 (0.38)	21.81	<.001	26.49 (1.12)

Abbreviations: PER = PERIOD protein; PR = percentage rhythm; ZT = zeitgeber Time; sLN_v_s = small lateral ventral neurons; lLN_v_s = large lateral ventral neurons; LN_d_s = lateral dorsal neurons; DN_1_ = dorsal neurons 1; DN_3_ = dorsal neurons 3. The single-component COSINOR based method was used to analyse time series of PER staining intensities in individual sub-groups of clock neurons to detect rhythmicity and estimate rhythm parameters. The statistic—PR estimates the strength of periodic component being tested in the analysis. The COSINOR being a regression-based method; PR is analogues to an R^2^ value (coefficient of determination) which represents percentage of overall variation explained by the fitted model. The *p-*values are from F-tests for the statistical significance of the period being tested.

Under T24, all clock neurons cycled almost in phase with each other, while the PER maxima occurred on average about 1.5 h earlier in *Pdf ^0^* mutants than in wild-type flies (Figure 5a). The maximum difference in phases of peak PER intensities among clock neurons was 1.03 h, range: ZT 2.37 h to ZT 3.40, standard deviation (SD) = 0.40 h, in wild-type flies, and 1.07 h (range: ZT 1.9 h to 2.97 h, SD = 0.42 h) in *Pdf ^0^* mutants. In wild-type flies, the DN_1_s had a slightly earlier phase than the rest of the clock neurons, which is consistent with earlier observations under long days (Menegazzi et al., 2013). In *Pdf ^0^* mutants, on the other hand, the s-LN_v_s had a later phase than the rest of the clock neurons (Figure 5a). Interestingly, the DN_1_s had a similar phase in wild-type flies and *Pdf ^0^* mutants.

Under T32, the clock neuron groups showed a larger variation in phase in both genotypes (Figures 4a and 5a, Table 6). The maximum phase difference among clock neuron was 4.96 h (range: ZT 26.27 h to 31.23 h, SD = 1.62 h) in wild-type flies and 4.69 h (range: ZT 24.44 h to 29.13 h, SD = 1.97 h) in *Pdf ^0^* mutants. The maximum phase difference among clock neurons in T32 was 4.81 times the difference under T24 in wild-type flies, and 4.38 times in *Pdf ^0^* mutants, respectively. This shows that the clock neurons lost synchrony as flies entrained to T32.

Under T32 the M neurons (s-LN_v_s) were delayed relative to the E neurons (5^th^ LN and LN_d_s) in both the genotypes (Figure 5a, Table 6). In wild-type flies, the M neurons peaked around lights-on, which was 3.4 h after the E neurons. In *Pdf ^0^* mutants, the M neurons peaked ~3 h before lights-on, which was ~4.5 h after the E neurons. These observations suggest that M and E neurons in *Pdf ^0^* mutants showed greater desynchrony than wild-type flies.

When comparing the phases of E neurons between wild-type flies and *Pdf ^0^* mutants under T32, it becomes clear that the E neurons peaked more than 3 h earlier in *Pdf ^0^* mutants than in wild-type flies (Figure 5a, Table 6). This suggests that the advanced phase of E neurons causes the early E activity in *Pdf ^0^* mutants under long zeitgeber cycles. As already found under T24, the DN_1_s showed no phase difference between wild-type flies and *Pdf ^0^* mutants. The same was true for the l-LN_v_.

When plotting the phases of the clock neurons as hours after lights-off (Figure 5b), it becomes obvious that the phase of the E neurons in wild-type flies did not change with zeitgeber period. It occurred ~12 h after lights-off under T24 and T32. Amazingly, this was not as precisely true for the phase of the E neurons in *Pdf ^0^* mutants, in which the phase of E activity phase locked to lights-off (Figure 2d-2f). Their PER peak occurred ~ 1 h earlier (~9 h after lights-off) under T32 than under T24 (~10 h after lights-off) (Figure 5b).

## Discussion

### Loss of PDF Alters Entrainment Properties of Activity Rhythms

The inability of *Pdf ^0^* flies to track changes in daylength is suggestive of defective entrainment mechanisms (Yoshii, Wülbeck, et al., 2009). We addressed this hypothesis using the T-cycle entrainment approach where we tested if the T-Ψ relationship of activity rhythm in *Pdf ^0^* mutants differ from wild-type flies. The phases of activity rhythms in both the genotypes systematically advanced with increasing period of T-cycle—a clear sign of entrainment. Comparison of T-Ψ relationships over T-cycle period range of 22-32 h showed that for every hour rise in T-cycle period, the E activity peak in *Pdf ^0^* flies advanced by much larger magnitude than in wildtype flies, clearly indicating altered light entrainment properties of *Pdf ^0^* mutants. As *Pdf ^0^* and wildtype flies showed the largest phase difference in their activity rhythm in T32, we tested whether they also exhibit a correlated phase difference in their molecular clock by studying PER rhythms in clock neurons. In the following, we discuss the importance of PDF for entrainment of *Drosophila* activity rhythms in the light of the present results on the molecular clock and observations from other studies.

### Desynchrony Among Clock Neurons Under T32

Consistent with previous studies, we observed synchrony among clock neurons in wildtype flies entrained to T24 (LD 16:8) (Figures 4a and 5a; Table 6) (Yoshii, Vanin, et al., 2009; Roberts et al., 2015). Only the DN_1_ had a slightly earlier phase than the other clock neurons, which is typical for long daylengths (Menegazzi et al., 2013). Under T32 (LD 16:16), wildtype flies exhibited robust 32 h PER rhythms in all the clock neurons, but unlike in T24, clock neuron groups showed phase differences of more than 4.5 h indicating desynchrony among them (Figures 4a and 5a). Pittendrigh and Minis (1972) speculated that entrainment of a circadian system comprising a network of clocks to non-24 h cycles would exhibit altered mutual phase relationships among constituent clocks compared to the phase relationships under 24 cycles. To the best of our knowledge, our result is a first direct evidence supporting the proposition of Pittendrigh and Minis (1972) in any invertebrate system. Phase differences between different groups of clock neurons entrained to the same zeitgeber cycle imply differences in their key entrainment properties that might be caused by different endogenous periods and/or by differences in phase-dependent light responsiveness. Indeed, the different groups of clock neurons are heterogenous with respect to their endogenous period (Blanchardon et al., 2001; Yoshii, Wülbeck, et al., 2009), their connections with other clock neurons (Hermann-Luibl and Helfrich-Förster, 2015; Yao and Shafer, 2014), and their light reception (Yoshii et al., 2015; Li et al., 2018; Helfrich-Förster, 2020).

Loss of PDF causes desynchrony among clock neurons under DD (Lin et al., 2004), however they remain apparently synchronous under LD 12:12. This observation lead Peng et al. (2003) to conclude that PDF mediated communication is not so important to maintain synchrony under entrained conditions, when individual clock cells can independently receive light inputs. Consistent with the observations in Peng et al. (2003), the clock neurons of *Pdf ^0^* mutants were almost in synchrony in our study under T24, although the phase relationship between neurons differed from that of wild-type flies (the s-LN_v_s peaked later than the other groups). We noticed an interesting pattern of desynchrony among the clock neurons under T32, which was similar in wildtype flies and *Pdf ^0^* mutants: the phases of s-LN_v_s (M neurons) were among the most delayed clock neurons, whereas 5^th^ LN and LN_d_s (E neurons) were among the earliest. In *Pdf ^0^* mutants, the phase difference between M and E neurons was larger indicating that the loss of PDF enhanced the desynchrony between M and E neurons (Figures 4a and 5a). Most likely, the absence of PDF also caused a slight desynchrony already under T24, which probably manifests as delayed phase of s-LN_v_s compared to the other clock neurons (Figures 4a and 5a). However, the phase differences between the other neuronal groups may simply be not large enough to be detected by a sampling interval of 4 h that is typically used in molecular studies. Our results thus demonstrate that the loss of PDF signaling desynchronizes clock neurons under entrained state in a similar way as it does under DD, which in turn suggests that the loss of PDF signaling alters light entrainment properties of *Drosophila* clock neurons.

In addition to desynchrony among clock neuron groups, the qualitative trends in PER waveform suggest that the loss of PDF may also induce a desynchrony within the individual clock neuron groups. The latter becomes evident when PER intensities of each neuron group are plotted as a mean of all the replicates instead of a scatterplot with COSINOR fit (Figure 4b). Figure 4b illustrates that the PER waveform of LN_d_s, DN_1_s and DN_3_s appears to be comprised of two peaks separated by a shallow trough. Although, one has to be cautious about this observation because our data are derived from a single experiment, it is worth to note that one peak approximately coincided with peak phase identified by COSINOR and the other occurred about 8 h later. The phase of the second later peak is almost identical with the PER peak phase in wildtype flies, suggesting that the loss of PDF advances only a subset of neurons within these groups. Within the LN_d_s, DN_1_s and DN_3_s only a subset of the neurons expresses the PDF receptor (Shafer et al., 2008; Mertens et al., 2005; Im and Taghert, 2010; Im et al., 2011), which probably explains why only a subset of these neurons are affected by the loss of PDF signaling. Most interestingly, the PDF receptor expressing neurons are identical with the CRY expressing clock neurons, while the other clock neurons are devoid of CRY (Im et al., 2011). CRY and PDF appear to interact and modulate the amplitude and phase of the clock neurons (Im et al., 2011). Together these observations may explain the altered phases of (a) clock neurons, (b) locomotor activity components under their control in the absence of PDF signaling; and consequently the abnormal light entrainment properties of locomotor activity rhythm in *Pdf ^0^* mutants.

### Loss of PDF Advances the Phase of E Neurons Under Entrained State

The LD12:12 and DD activity phenotypes of *Pdf ^0^* and PDF receptor null mutants *pdfr*^han5304^ are identical to flies with ablation or electrical silencing of PDF expressing neurons (Renn et al., 1999; Wu et al., 2008; Lear et al., 2005). These observations show that the effect of loss of PDF signaling is equivalent to loss of PDF expressing neurons which suggests that locomotor activity in *Pdf ^0^* mutants is primarily driven by a subset of non-PDF neurons (Renn et al., 1999; Lin et al., 2004). As the E activity peak is the only prominent activity bout in *Pdf ^0^* mutants, E neurons may be regarded as primary driver of locomotor activity in these flies. The advanced phase of the molecular clock in LN_d_s (a subset of E neurons) of *Pdf ^0^* flies after several days in DD suggest a shorter intrinsic period of LN_d_s in the absence of PDF signaling (Lin et al., 2004). In wildtype flies, the s-LN_v_s reset (delay) the phase of molecular clock in LN_d_s via PDF signaling (Stoleru et al., 2005; Wu et al., 2008; Guo et al., 2014; Seluzicki et al., 2014), while E activity in *Pdf ^0^* flies remains slightly advanced relative to wildtype flies under LD 12:12 (Renn et al., 1999). All these observations suggest that PDF is needed for daily resetting of E neurons by delaying their phase, and thus predict the advanced phase of E neurons under LD 12:12 in the absence of PDF signaling. Consistent with this prediction, Lear et al. (2009) indeed found a subtle advance of E neurons in *pdfr*^han5304^ mutant as compared to wild-type flies, but indicated a need for higher sampling resolution to conclusively demonstrate a molecular phase shift in the E neurons. Our T32 experiments clearly demonstrate that the E neurons of *Pdf ^0^* mutants are advanced by more than 3 h relative to the E neurons of wildtype flies (Figures 4a and 5a; Table 6). This suggests that the loss of PDF advances the phase of E neurons, which in turn advances the phase of E activity. However, we observed that the E activity peak occurred about ~7 h earlier in *Pdf ^0^* mutants than in wildtype, which cannot be explained by the ~3 h advance of the E neurons alone. The results of Liang et al. (2016, 2017) may explain this discrepancy, because these authors found that the PDF signaling—(1) delays the phase of neural activity rhythms in E neurons, and (2) partially modulates light induced phase shifts in neural activity rhythms through a mechanism apparently independent of the effects of PDF on the phase of molecular clock. Therefore, we speculate that the ~7 h advanced phase of E activity in *Pdf^0^* mutants relative to wildtype reflects the combined action of PDF’s phase delaying effects on molecular clock and additional effects on the neural activity rhythms of E neurons. Moreover, these explanations also corroborate our observation that the shorter period is insufficient to explain the advanced phase of E activity in *Pdf ^0^* mutants (Figure 3; Table 2). We also observed that sLN_v_s of *Pdf ^0^* mutants were advanced by more than 2 h relative to those of wildtype flies under T32 suggesting that PDF also delays the phase of M neurons under entrained state; however, the phase of sLN_v_s may be of little functional significance as PDF expressing neurons apparently lack control over locomotor activity in *Pdf^0^* mutants (Renn et al., 1999; Lin et al., 2004).

### Phase Locking of E Neurons to Lights-Off Transition

We observed that the activity peak in *Pdf*^0^ mutants lagged behind the lights-off transition by a constant time interval in all the T-cycles, or in other words, phase locked to the lights-off transition (right side panels in Figures 1 and 2d-2f). This suggests that the clock(s) regulating activity rhythms in *Pdf*^0^ mutants behave like an hourglass based system. Such hourglass clocks are assumed to be completely damped by the end of light phase and start afresh at the beginning of each night to drive a rhythmic output that then appears phase locked to lights-off (Pittendrigh and Minis, 1964; Pittendrigh, 1966). Consequently, the phase of such a rhythm under T-cycle entrainment would not change with T-cycle period (Roenneberg et al., 2005), exactly as we observed for *Pdf*^0^ mutants. As the activity peak in *Pdf*^0^ mutants appears to be primarily regulated by E neurons, we tested if the phases of E neurons in *Pdf* ^0^ mutants are also locked to the lights-off transition. At a 16 h daylength, the activity of *Pdf* ^0^ mutants peaked about 19 h after the lights-off transition across all T cycles (Figure 5b). For PER cycling in the E neurons, the phase-locking was less perfect under these conditions: in the 5^th^ LN_v_ and LN_d_s, PER peaked 10.13 h after lights-off under T24, and 8.44 h/ 9.16 h after lights-off under T32. Since this is only 1 to 1.5 h earlier than expected under a perfect phase locking we may still regard the E neurons of *Pdf* ^0^ mutant as phase locked to lights-off, which supports the hypothesis that the molecular clocks regulating activity rhythm in *Pdf* ^0^ mutants behave as hour-glasses. Interestingly, the examination of wildtype data showed that although activity rhythm did not phase lock to lights-off, E neurons of wildtype flies showed clear evidence for phase locking to the lights-off transition. This phase locking appeared even better than that of *Pdf* ^0^ mutants as PER peaked always about 12 h after lights-off (11.34 h and 11.35 h under T24, 11.83 and 12.22 h under T32 in 5^th^ LN and LN_d_s respectively; Figure 5b). If E neurons in *Pdf* ^0^ mutant as well as in wildtype flies are phase locked to lights-off transition, it is puzzling that the activity rhythm of wildtype flies is not phase locked to lights-off. A possible explanation for this discrepancy is again the phase-delaying effect of PDF on E-neuron activity, which is absent in *Pdf* ^0^ mutants (Liang et al., 2016, 2017). We therefore speculate that despite the phase locking of molecular clocks in E neurons to lights-off transition, the E activity of wild-type flies does not phase lock to lights-off due to the additional phase delaying effects of PDF. In *Pdf* ^0^ mutants, on the other hand, E activity faithfully reflects the phase of E neurons that are phase locked to lights-off transition.

The phase locking of the E neurons to lights-off in *Pdf* ^0^ mutants as well as wildtype flies reveals an hourglass like behaviour of E neurons. However, rhythmic nature of E neurons under DD in the absence of PDF (Lin et al., 2004) or s-LN_v_ molecular clock (Grima et al., 2004) suggests that the hourglass-like behaviour of E neurons is unlikely to arise from a simple light-driven molecular clock. The *Drosophila* visual photoreceptors are particularly important for differential light sensitivity of M and E oscillators that allows tracking the changes in photo-period (Yoshii et al., 2004; Rieger et al., 2006; Kistenpfennig et al., 2018). Perhaps, the photic inputs to E neurons from the visual photoreceptors that help flies set the phase of evening activity in wake of changes in daily photo-period may explain the phase locking of E neurons to lights-off transition in our T-cycle experiments.

In summary, our results demonstrate that PDF is necessary to maintain synchrony among clock neurons under LD cycles like under DD, and to delay the phase of E neurons. These results in the light of observations from previous studies show that PDF is necessary for appropriate timing of E activity by delaying the phases of molecular clock and neural activity rhythm of E neurons. Together, our results suggest that the PDF is necessary for appropriate phase relationship of *Drosophila* activity rhythms and it plays a more complex role than thought before. Our analysis also shows that the phase of molecular clock in E neurons is set by lights-off transition, revealing a distinct entrainment property of E neurons.

## Supplemental Material

sj-tif-1-jbr-10.1177_07487304211032336 – Supplemental material for The Neuropeptide PDF Is Crucial for Delaying the Phase of Drosophila’s Evening Neurons Under Long Zeitgeber Periods Click here for additional data file. Supplemental material, sj-tif-1-jbr-10.1177_07487304211032336 for The Neuropeptide PDF Is Crucial for Delaying the Phase of Drosophila’s Evening Neurons Under Long Zeitgeber Periods by Koustubh M. Vaze and Charlotte Helfrich-Förster in Journal of Biological Rhythms

sj-tif-2-jbr-10.1177_07487304211032336 – Supplemental material for The Neuropeptide PDF Is Crucial for Delaying the Phase of Drosophila’s Evening Neurons Under Long Zeitgeber Periods Click here for additional data file. Supplemental material, sj-tif-2-jbr-10.1177_07487304211032336 for The Neuropeptide PDF Is Crucial for Delaying the Phase of Drosophila’s Evening Neurons Under Long Zeitgeber Periods by Koustubh M. Vaze and Charlotte Helfrich-Förster in Journal of Biological Rhythms

sj-tif-3-jbr-10.1177_07487304211032336 – Supplemental material for The Neuropeptide PDF Is Crucial for Delaying the Phase of Drosophila’s Evening Neurons Under Long Zeitgeber Periods Click here for additional data file. Supplemental material, sj-tif-3-jbr-10.1177_07487304211032336 for The Neuropeptide PDF Is Crucial for Delaying the Phase of Drosophila’s Evening Neurons Under Long Zeitgeber Periods by Koustubh M. Vaze and Charlotte Helfrich-Förster in Journal of Biological Rhythms

sj-tif-4-jbr-10.1177_07487304211032336 – Supplemental material for The Neuropeptide PDF Is Crucial for Delaying the Phase of Drosophila’s Evening Neurons Under Long Zeitgeber Periods Click here for additional data file. Supplemental material, sj-tif-4-jbr-10.1177_07487304211032336 for The Neuropeptide PDF Is Crucial for Delaying the Phase of Drosophila’s Evening Neurons Under Long Zeitgeber Periods by Koustubh M. Vaze and Charlotte Helfrich-Förster in Journal of Biological Rhythms

sj-tif-5-jbr-10.1177_07487304211032336 – Supplemental material for The Neuropeptide PDF Is Crucial for Delaying the Phase of Drosophila’s Evening Neurons Under Long Zeitgeber Periods Click here for additional data file. Supplemental material, sj-tif-5-jbr-10.1177_07487304211032336 for The Neuropeptide PDF Is Crucial for Delaying the Phase of Drosophila’s Evening Neurons Under Long Zeitgeber Periods by Koustubh M. Vaze and Charlotte Helfrich-Förster in Journal of Biological Rhythms

## References

[bibr1-07487304211032336] Abhilash L Sheeba V (2019) RhythmicAlly: your R and Shiny-based open-source ally for the analysis of biological rhythms. J Biol Rhythms 34:551-561. 31307268 10.1177/0748730419862474

[bibr2-07487304211032336] Aschoff J Wever R (1962) On phase relationships between periods of biological rhythms and of zeitgebers. Z vergl Physiol 46:115-128.

[bibr3-07487304211032336] Blanchardon E Grima B Klarsfeld A Chélot E Hardin PE Préat T Rouyer F (2001) Defining the role of *Drosophila* lateral neurons in the control of circadian rhythms in motor activity and eclosion by targeted genetic ablation and PERIOD protein overexpression. Eur J Neurosci 13:871-888. 11264660 10.1046/j.0953-816x.2000.01450.x

[bibr4-07487304211032336] Cornelissen G (2014) Cosinor-based rhythmometry. Theor Biol Med Model 11:16. 24725531 10.1186/1742-4682-11-16 PMC3991883

[bibr5-07487304211032336] Grima B Chélot E Xia R Rouyer F (2004) Morning and evening peaks of activity rely on different clock neurons of the *Drosophila* brain. Nature 431:869-873. 15483616 10.1038/nature02935

[bibr6-07487304211032336] Guo F Cerullo I Chen X Rosbash M (2014) PDF neuron firing phase-shifts key circadian activity neurons in *Drosophila*. eLife 3:e02780. 10.7554/eLife.02780 PMC4092873 24939987

[bibr7-07487304211032336] Helfrich C Engelmann W (1987) Evidences for circadian rhythmicity in the *per^o^* mutant of *Drosophila melanogaster*. Z Naturforsch 42c:1335-1338. 10.1515/znc-1987-11-1231 2966505

[bibr8-07487304211032336] Helfrich-Förster C (1995) The *period* clock gene is expressed in central nervous system neurons which also produce a neuropeptide that reveals the projections of circadian pacemaker cells within the brain of *Drosophila melanogaster*. Proc Natl Acad Sci U S A 92:612-616. 7831339 10.1073/pnas.92.2.612 PMC42792

[bibr9-07487304211032336] Helfrich-Förster C (2001) The locomotor activity rhythm of *Drosophila melanogaster* is controlled by a dual oscillator system. J Insect Physiol 47:877-887.

[bibr10-07487304211032336] Helfrich-Förster C (2003) The neuroarchitecture of the circadian clock in the brain of *Drosophila melanogaster*. Microsc Res Tech 62:94-102. 12966496 10.1002/jemt.10357

[bibr11-07487304211032336] Helfrich-Förster C (2020) Light input pathways to the circadian clock of insects with an emphasis on the fruit fly *Drosophila melanogaster*. J Comp Physiol A Neuroethol Sens Neural Behav Physiol 206:259-272. 31691095 10.1007/s00359-019-01379-5 PMC7069913

[bibr12-07487304211032336] Helfrich-Förster C Shafer OT Wülbeck C Grieshaber E Rieger D Taghert P (2007) Development and morphology of the clock-gene-expressing lateral neurons of *Drosophila melanogaster*. J Comp Neurol 500:47-70. 17099895 10.1002/cne.21146

[bibr13-07487304211032336] Helfrich-Förster C Yoshii T Wülbeck C Grieshaber E Rieger D Bachleitner W Cusumano P Rouyer F (2007) The lateral and dorsal neurons of *Drosophila melanogaster*: new insights about their morphology and function. Cold Spring Harb Symp Quant Biol 72:517-525. 18419311 10.1101/sqb.2007.72.063

[bibr14-07487304211032336] Hermann-Luibl C Helfrich-Förster C (2015) Clock network in *Drosophila*. Curr Opin Insect Sci 7:65-70. 32846682 10.1016/j.cois.2014.11.003

[bibr15-07487304211032336] Hoffmann K (1968) On the influence of zeitgeber strength on the phase relationship of the entrained circadian clock. Z Vergl Physiol 62:93-110.

[bibr16-07487304211032336] Horn M Mitesser O Hovestadt T Yoshii T Rieger D Helfrich-Förster C (2019) The circadian clock improves fitness in the fruit fly, *Drosophila melanogaster*. Front Physiol 10:1374. 10.3389/fphys.2019.01374 PMC6838225 31736790

[bibr17-07487304211032336] Im SH Taghert PH (2010) PDF receptor expression reveals direct interactions between circadian oscillators in *Drosophila*. J Comp Neurol 518:1925-1945. 20394051 10.1002/cne.22311 PMC2881544

[bibr18-07487304211032336] Im SH Li W Taghert PH (2011) PDFR and CRY signaling converge in a subset of clock neurons to modulate the amplitude and phase of circadian behavior in *Drosophila*. PLoS ONE 6:e18974. 21559487 10.1371/journal.pone.0018974 PMC3084726

[bibr19-07487304211032336] Johnson CH (1992) Phase response curves: what can they tell us about circadian clocks? In: Hiroshige T Honma K editors. Circadian Clocks from Cell to Human. Sapporo (Japan): Hokkaido University Press, p. 209-249.

[bibr20-07487304211032336] Kaneko M Helfrich-Förster C Hall JC (1997) Spatial and temporal expression of the period and timeless genes in the developing nervous system of *Drosophila*: newly identified pacemaker candidates and novel features of clock gene product cycling. J Neurosci 20:3339–3353. 10.1523/JNEUROSCI.17-17-06745.1997 PMC6573141 9254686

[bibr21-07487304211032336] Kistenpfennig C Nakayama M Nihara R Tomioka K Helfrich-Förster C Yoshii T (2018) A tug-of-war between cryptochrome and the visual system allows the adaptation of evening activity to long photoperiods in *Drosophila melanogaster*. J Biol Rhythms 33:24-34. 29179610 10.1177/0748730417738612

[bibr22-07487304211032336] Konopka RJ Benzer S (1971) Clock mutants of *Drosophila melanogaster*. Proc Natl Acad Sci U S A 68:2112-2116. 5002428 10.1073/pnas.68.9.2112 PMC389363

[bibr23-07487304211032336] Lear BC Merrill CE Lin JM Schroeder A Zhang L Allada R (2005) A G protein-coupled receptor, groom-of-PDF, is required for PDF neuron action in circadian behavior. Neuron 48:221-227. 16242403 10.1016/j.neuron.2005.09.008

[bibr24-07487304211032336] Lear BC Zhang L Allada R (2009) The neuropeptide PDF acts directly on evening pacemaker neurons to regulate multiple features of circadian behavior. PLoS Biol 7:e1000154. 19621061 10.1371/journal.pbio.1000154 PMC2702683

[bibr25-07487304211032336] Lee Gierke C Cornelissen G (2016) Chronomics analysis toolkit (CATkit). Biol Rhythm Res 47:163-181.

[bibr26-07487304211032336] Li MT Cao LH Xiao N Tang M Deng B Yang T Yoshii T Luo DG (2018) Hub- organized parallel circuits of central circadian pacemaker neurons for visual photoentrainment in *Drosophila*. Nat Commun 9:4247. 30315165 10.1038/s41467-018-06506-5 PMC6185921

[bibr27-07487304211032336] Liang X Holy TE Taghert PH (2016) Synchronous *Drosophila* circadian pacemakers display nonsynchronous Ca^2+^ rhythms in vivo. Science 351:976-981. 26917772 10.1126/science.aad3997 PMC4836443

[bibr28-07487304211032336] Liang X Holy TE Taghert PH (2017) A series of suppressive signals within the *Drosophila* circadian neural circuit generates sequential daily outputs. Neuron 94:1173-1189. 28552314 10.1016/j.neuron.2017.05.007 PMC5502710

[bibr29-07487304211032336] Lin Y Stormo GD Taghert PH (2004) The neuropeptide pigment-dispersing factor coordinates pacemaker interactions in the *Drosophila* circadian system. J Neurosci 24:7951-7957. 15356209 10.1523/JNEUROSCI.2370-04.2004 PMC6729918

[bibr30-07487304211032336] Menegazzi P Vanin S Yoshii T Rieger D Hermann C Dusik V Kyriacou CP Helfrich-Förster C Costa R (2013) *Drosophila* clock neurons under natural conditions. J Biol Rhythms 28:3-14. 23382587 10.1177/0748730412471303

[bibr31-07487304211032336] Mertens I Vandingenen A Johnson EC Shafer OT Li W Trigg JS De Loof A Schoofs L Taghert PH (2005) PDF receptor signaling in *Drosophila* contributes to both circadian and geotactic behaviors. Neuron 48:213-219. 16242402 10.1016/j.neuron.2005.09.009

[bibr32-07487304211032336] Peng Y Stoleru D Levine JD Hall JC Rosbash M (2003) *Drosophila* free-running rhythms require intercellular communication. PLoS Biol 1:E13. 12975658 10.1371/journal.pbio.0000013 PMC193604

[bibr33-07487304211032336] Picot M Cusumano P Klarsfeld A Ueda R Rouyer F (2007) Light activates output from evening neurons and inhibits output from morning neurons in the *Drosophila* circadian clock. PLoS Biol 5:2513-2521. 10.1371/journal.pbio.0050315 PMC2229858 18044989

[bibr34-07487304211032336] Pittendrigh CS (1966) The circadian oscillation in *Drosophila* pseudoobscura pupae: a model for the photoperiodic clock. Z Pflanzenphysiol 54:275-307.

[bibr35-07487304211032336] Pittendrigh CS Minis DH (1964) The entrainment of circadian oscillations by light and their role as photoperiodic clocks. Am Nat 98:261-294.

[bibr36-07487304211032336] Pittendrigh CS Minis DH (1972) Circadian systems: longevity as a function of circadian resonance in *Drosophila melanogaster*. Proc Natl Acad Sci U S A 69:1537-1539. 4624759 10.1073/pnas.69.6.1537 PMC426743

[bibr37-07487304211032336] Renn SC Park JH Rosbash M Hall JC Taghert PH (1999) A *pdf* neuropeptide gene mutation and ablation of PDF neurons each cause severe abnormalities of behavioral circadian rhythms in *Drosophila*. Cell 99:791-802. 10619432 10.1016/s0092-8674(00)81676-1

[bibr38-07487304211032336] Rieger D Shafer OT Tomioka K Helfrich-Förster C (2006) Functional analysis of circadian pacemaker neurons in *Drosophila melanogaster*. J Neurosci 26:2531-2543. 16510731 10.1523/JNEUROSCI.1234-05.2006 PMC6793667

[bibr39-07487304211032336] Roberts L Leise TL Noguchi T Galschiodt AM Houl JH Welsh DK Holmes TC (2015) Light evokes rapid circadian network oscillator desynchrony followed by gradual phase retuning of synchrony. Curr Biol 25:858-867. 25754644 10.1016/j.cub.2015.01.056 PMC4399721

[bibr40-07487304211032336] Roenneberg T Daan S Merrow M (2003) The art of entrainment. J Biol Rhythms 18:183-194. 12828276 10.1177/0748730403018003001

[bibr41-07487304211032336] Roenneberg T Dragovic Z Merrow M (2005) Demasking biological oscillators: properties and principles of entrainment exemplified by the Neurospora circadian clock. Proc Natl Acad Sci U S A 102:7742-7747. 15899977 10.1073/pnas.0501884102 PMC1140435

[bibr42-07487304211032336] Ruben M Drapeau MD Mizrak D Blau J (2012) A mechanism for circadian control of pacemaker neuron excitability. J Biol Rhythms 27:353-364. 23010658 10.1177/0748730412455918 PMC4019749

[bibr43-07487304211032336] Schmid B Helfrich-Förster C Yoshii T (2011) A new ImageJ plug-in ‘ActogramJ’ for chronobiological analyses. J Biol Rhythms 26:464-467. 21921300 10.1177/0748730411414264

[bibr44-07487304211032336] Schubert FK Hagedorn N Yoshii T Helfrich-Förster C Rieger D (2018) Neuroanatomical details of the lateral neurons of *Drosophila melanogaster* support their functional role in the circadian system. J Comp Neurol 526:1209-1231. 29424420 10.1002/cne.24406 PMC5873451

[bibr45-07487304211032336] Seluzicki A Flourakis M Kula-Eversole E Zhang L Kilman V Allada R (2014) Dual PDF signalling pathways reset clocks via TIMELESS and acutely excite target neurons to control circadian behavior. PLoS Biol 12:e1001810. 24643294 10.1371/journal.pbio.1001810 PMC3958333

[bibr46-07487304211032336] Shafer OT Kim DJ Dunbar-Yaffe R Nikolaev VO Lohse MJ Taghert PH (2008) Widespread receptivity to neuropeptide PDF throughout the neuronal circadian clock network of *Drosophila* revealed by real-time cyclic AMP imaging. Neuron 58:223-237. 18439407 10.1016/j.neuron.2008.02.018 PMC2586874

[bibr47-07487304211032336] Stoleru D Nawathean P Fernández MP Menet JS Ceriani MF Rosbash M (2007) The *Drosophila* circadian network is a seasonal timer. Cell 129:207-219. 17418796 10.1016/j.cell.2007.02.038

[bibr48-07487304211032336] Stoleru D Peng Y Agosto J Rosbash M (2004) Coupled oscillators control morning and evening locomotor behaviour of *Drosophila*. Nature 431:862-868. 15483615 10.1038/nature02926

[bibr49-07487304211032336] Stoleru D Peng Y Nawathean P Rosbash M (2005) A resetting signal between *Drosophila* pacemakers synchronizes morning and evening activity. Nature 438:238-242. 16281038 10.1038/nature04192

[bibr50-07487304211032336] Top D Young MW (2018) Coordination between differentially regulated circadian clocks generates rhythmic behavior. Cold Spring Harb Perspect Biol 10:a033589. 28893860 10.1101/cshperspect.a033589 PMC6028074

[bibr51-07487304211032336] Wu Y Cao G Nitabach MN (2008) Electrical silencing of PDF neurons advances the phase of non-PDF clock neurons in *Drosophila*. J Biol Rhythms 23:117-128. 18375861 10.1177/0748730407312984

[bibr52-07487304211032336] Yao Z Shafer OT (2014) The *Drosophila* circadian clock is a variably coupled network of multiple peptidergic units. Science 343:1516-1520. 24675961 10.1126/science.1251285 PMC4259399

[bibr53-07487304211032336] Yoshii T Funada Y Ibuki-Ishibashi T Matsumoto A Tanimura T Tomioka K (2004) *Drosophila cry^b^* mutation reveals two circadian clocks that drive locomotor rhythm and have different responsiveness to light. J Insect Physiol 50:479-488. 15183277 10.1016/j.jinsphys.2004.02.011

[bibr54-07487304211032336] Yoshii T Hermann-Luibl C Helfrich-Förster C (2015) Circadian light-input pathways in *Drosophila*. Commun Integr Biol 9:e1102805. 27066180 10.1080/19420889.2015.1102805 PMC4802797

[bibr55-07487304211032336] Yoshii T Vanin S Costa R Helfrich-Förster C (2009) Synergic entrainment of *Drosophila*’s circadian clock by light and temperature. J Biol Rhythms 24:452-464. 19926805 10.1177/0748730409348551

[bibr56-07487304211032336] Yoshii T Wülbeck C Sehadova H Veleri S Bichler D Stanewsky R Helfrich-Förster C (2009) The neuropeptide *pigment-dispersing factor* adjusts period and phase of *Drosophila*’s clock. J Neurosci 29:2597-2610. 19244536 10.1523/JNEUROSCI.5439-08.2009 PMC6666242

