# Supplementary figures and images for: The Neuropeptide PDF Is Crucial for Delaying the Phase of Drosophila’s Evening Neurons Under Long Zeitgeber Periods
Source: J Biol Rhythms. 2021 Aug 24;36(5):442–60. doi: 10.1177/07487304211032336 (PMC8442139; doi:10.1177/07487304211032336)

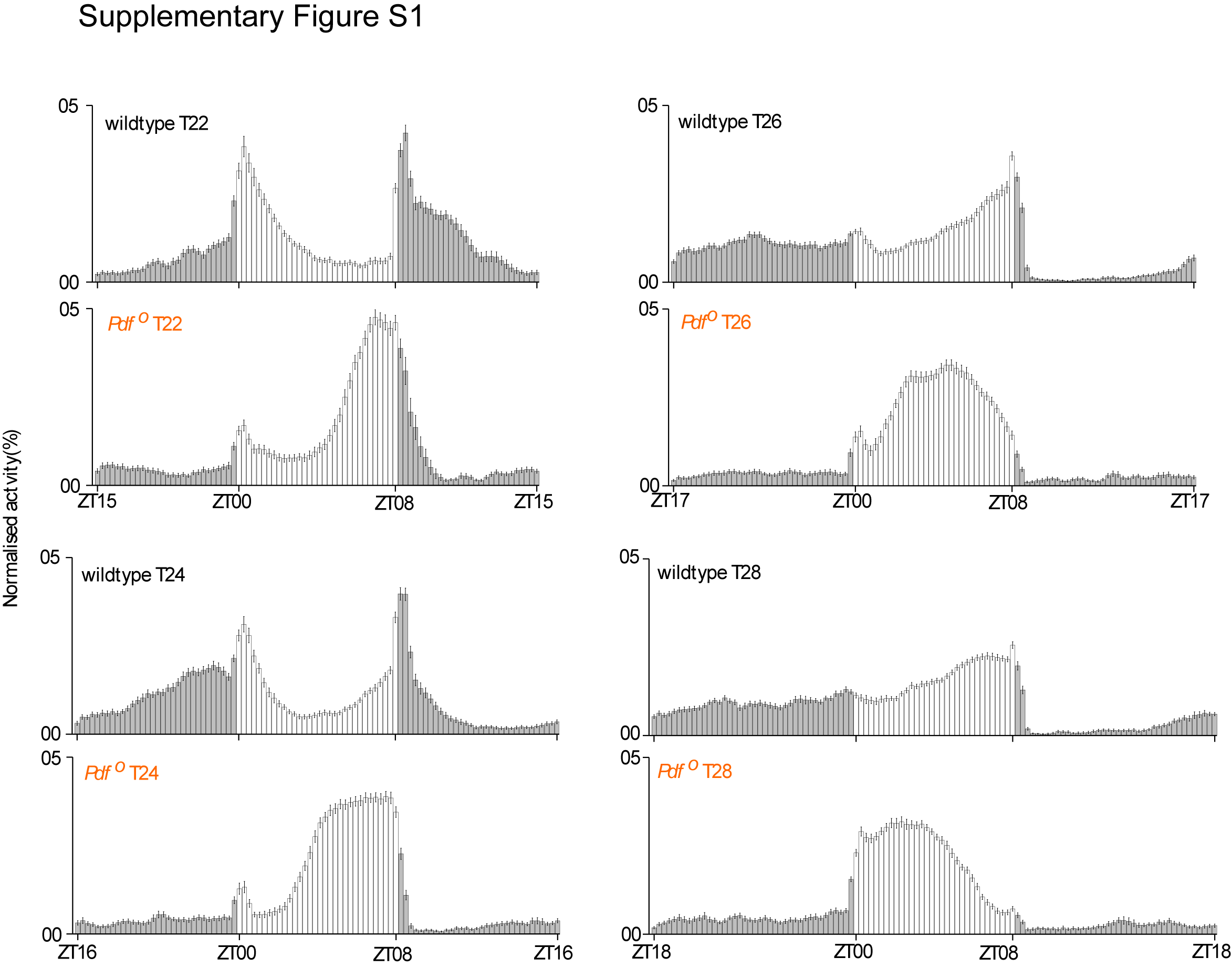

Supplement: sj-tif-1-jbr-10.1177_07487304211032336 – Supplemental material for The Neuropeptide PDF Is Crucial for Delaying the Phase of Drosophila’s Evening Neurons Under Long Zeitgeber Periods [file sj-tif-1-jbr-10.1177_07487304211032336.tif]

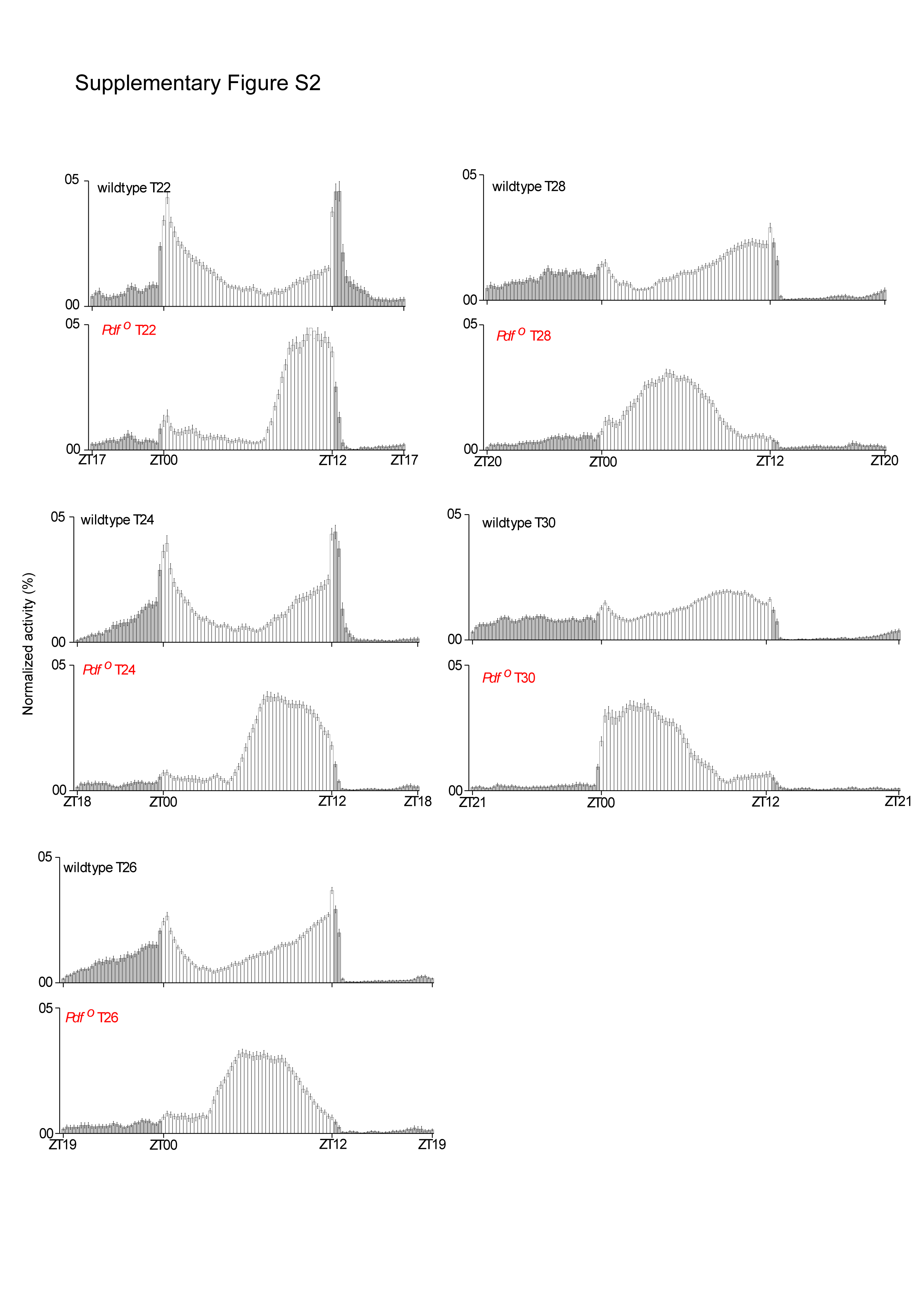

Supplement: sj-tif-2-jbr-10.1177_07487304211032336 – Supplemental material for The Neuropeptide PDF Is Crucial for Delaying the Phase of Drosophila’s Evening Neurons Under Long Zeitgeber Periods [file sj-tif-2-jbr-10.1177_07487304211032336.tif]

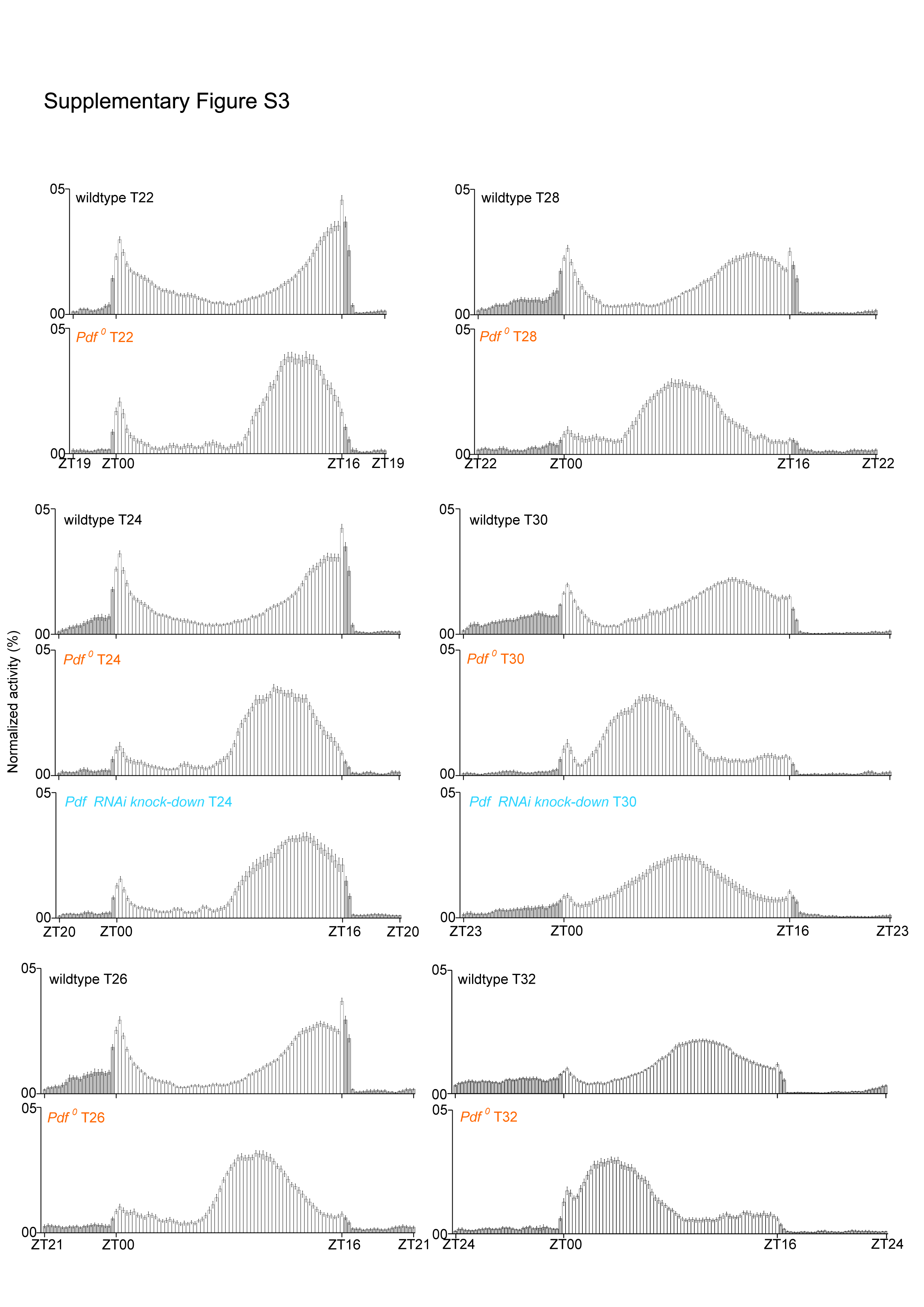

Supplement: sj-tif-3-jbr-10.1177_07487304211032336 – Supplemental material for The Neuropeptide PDF Is Crucial for Delaying the Phase of Drosophila’s Evening Neurons Under Long Zeitgeber Periods [file sj-tif-3-jbr-10.1177_07487304211032336.tif]

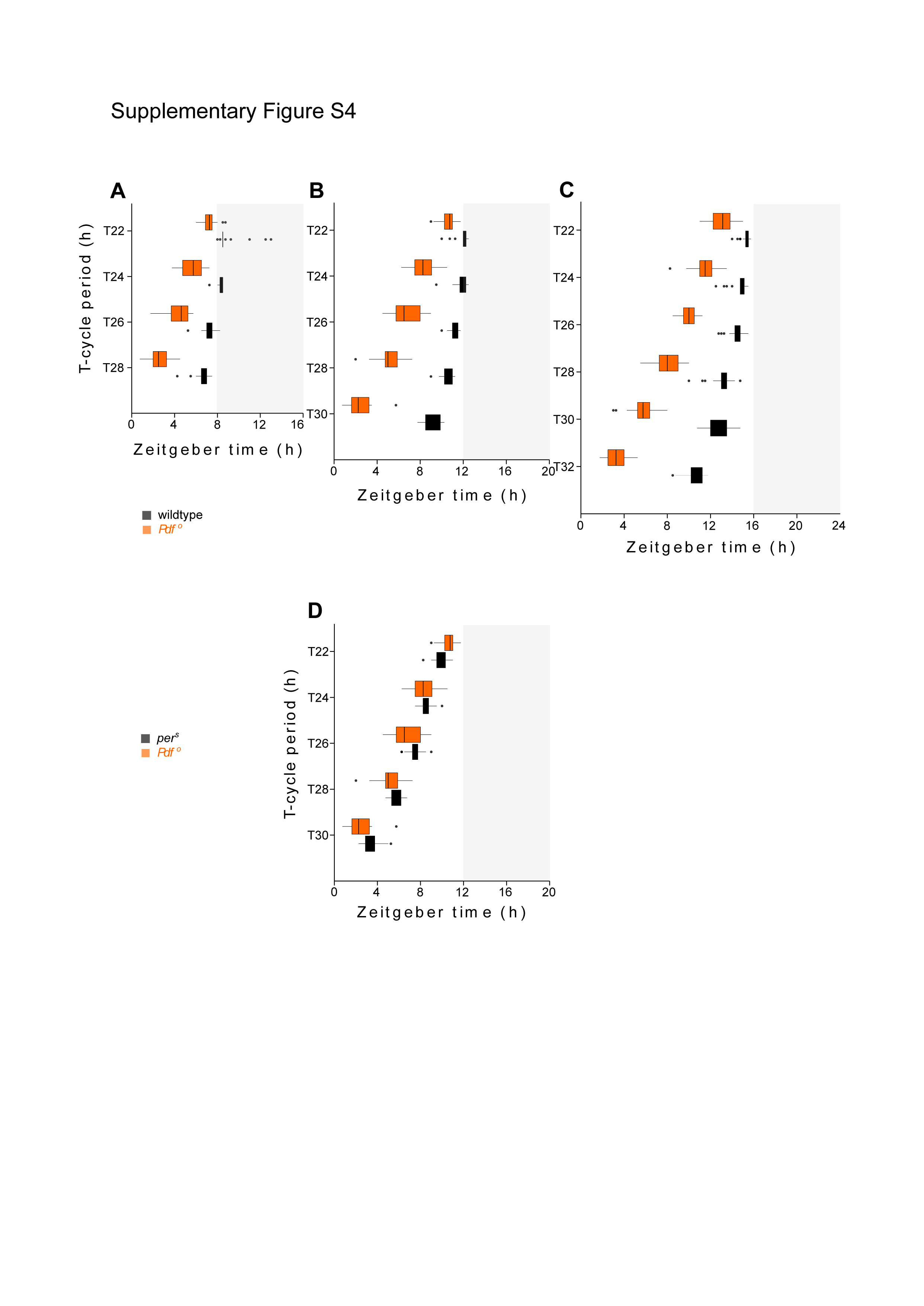

Supplement: sj-tif-4-jbr-10.1177_07487304211032336 – Supplemental material for The Neuropeptide PDF Is Crucial for Delaying the Phase of Drosophila’s Evening Neurons Under Long Zeitgeber Periods [file sj-tif-4-jbr-10.1177_07487304211032336.tif]

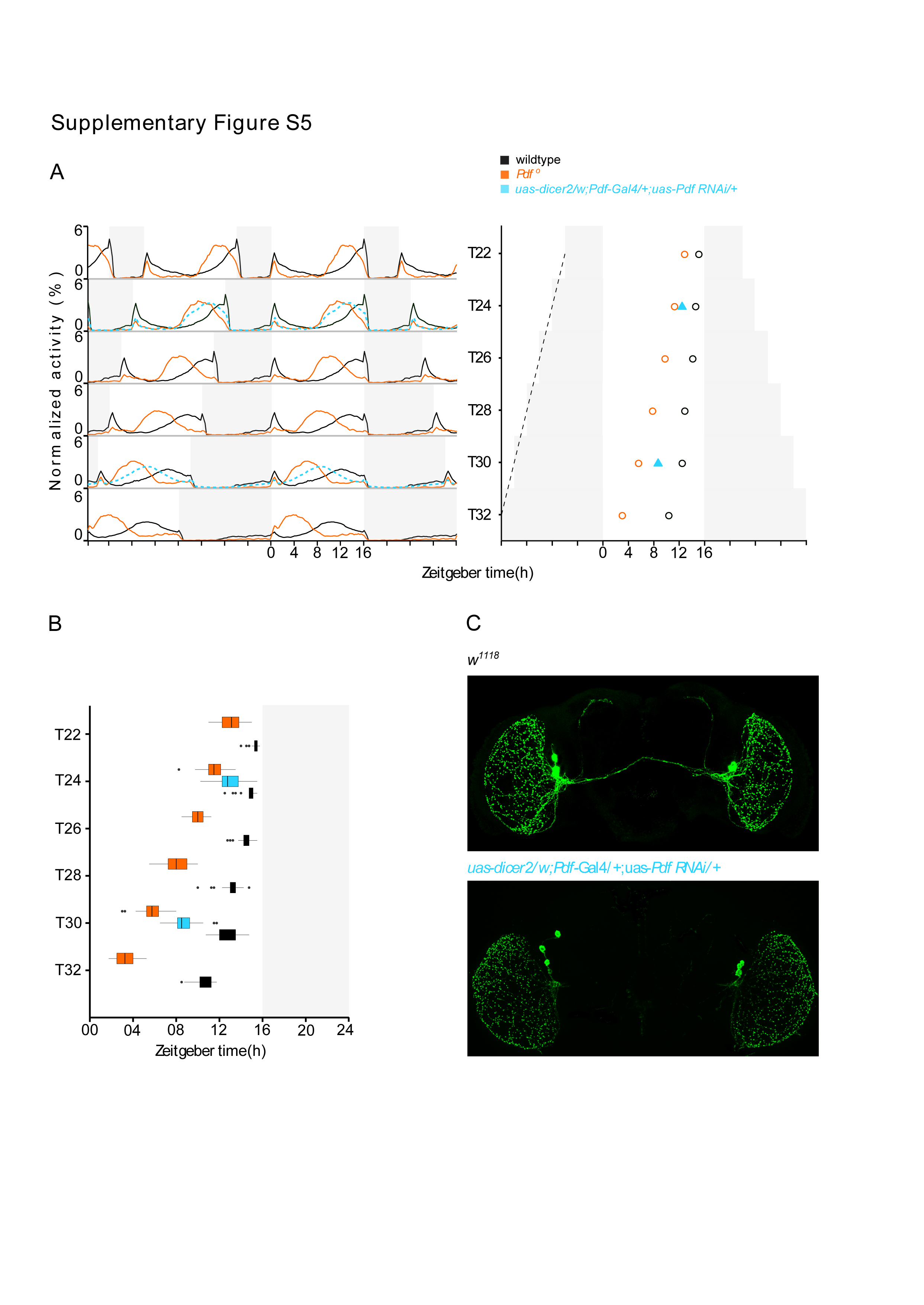

Supplement: sj-tif-5-jbr-10.1177_07487304211032336 – Supplemental material for The Neuropeptide PDF Is Crucial for Delaying the Phase of Drosophila’s Evening Neurons Under Long Zeitgeber Periods [file sj-tif-5-jbr-10.1177_07487304211032336.tif]
